# Polyoxometalate‐Bridged Synthesis of Superstructured Mesoporous Polymers and Their Derivatives for Sodium–Iodine Batteries

**DOI:** 10.1002/advs.202301918

**Published:** 2023-04-25

**Authors:** Tingting Zhang, Facai Wei, Yong Wu, Wenda Li, Lingyan Huang, Jianwei Fu, Chengbin Jing, Jiangong Cheng, Shaohua Liu

**Affiliations:** ^1^ State Key Laboratory of Precision Spectroscopy Engineering Research Center of Nanophotonics and Advanced Instrument Ministry of Education School of Physics and Electronic Science East China Normal University Shanghai 200241 P. R. China; ^2^ School of Materials Science and Engineering Zhengzhou University 75 Daxue Road Zhengzhou 450052 P. R. China; ^3^ State Key Lab of Transducer Technology, Shanghai Institute of Microsystem and Information Technology Chinese Academy of Sciences 200050 Shanghai P. R. China

**Keywords:** mesoporous polymers, Na—I_2_ batteries, polyoxometalate, self‐assembly, soft templates

## Abstract

Despite the impressive progress in mesoporous materials over past decades, for those precursors having no well‐matched interactions with soft templates, there are still obstacles to be guided for mesoporous structure via soft‐template strategies. Here, a polyoxometalate‐assisted co‐assembly route is proposed for controllable construction of superstructured mesoporous materials by introducing polyoxometalates as bifunctional bridge units, which weakens the self‐nucleation tendency of the precursor through coordination interactions and simultaneously connects the template through the induced dipole–dipole interaction. By this strategy, a series of meso‐structured polymers, featuring highly open radial mesopores and dendritic pore walls composed of continuous interwoven nanosheets can be facilely obtained. Further carbonization gave rise to nitrogen‐doped hierarchical mesoporous carbon decorated uniformly with ultrafine *γ*‐Mo_2_N nanoparticles. Density functional theory proves that nitrogen‐doped carbon and *γ*‐Mo_2_N can strongly adsorb polyiodide ions, which effectively alleviate polyiodide dissolving in organic electrolytes. Thereby, as the cathode materials for sodium–iodine batteries, the I_2_‐loaded carbonaceous composite shows a high specific capacity (235 mA h g^−1^ at 0.5 A g^−1^), excellent rate performance, and cycle stability. This work will open a new venue for controllable synthesis of new hierarchical mesoporous functional materials, and thus promote their applications toward diverse fields.

## Graphical abstract

A novel polyoxometalates‐assisted co‐assembly strategy to synthesize polymer nanosphere with uniform size, highly wrinkled pore walls, and open radial mesoporous pores is proposed. Theoretical and experimental results prove that the mesoporous structure and hybrid composition can synergistically improve its electrochemical performance as a cathode for Na—I_2_ batteries.

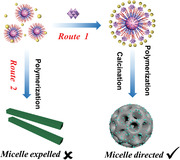

## 1 Introduction

By virtue of interconnected pore networks, exposed reaction sites, and high accessible surfaces, mesoporous materials have attracted extensive attention in various fields since their advent.^[^ ^1^, ^2^, ^3^, ^4^ ^]^ The soft template–directed synthesis technologies have contributed to fabricating a series of mesoporous materials with various components and nanoarchitectures.^[^ ^5^, ^6^, ^7^, ^8^ ^]^ However, this soft template strategy highly depends on the well‐matched forces between surfactant molecules and precursors to form mesoscopic structures by their co‐assembly process.^[^ ^9^ ^]^ Therefore, for some precursors that tend to self‐stack/self‐nucleate and grow into a continuous macroscopic phase and thus expel soft templates from final bulk crystals, the introduction of mesoporous structures is still hampered.^[^ ^10^, ^11^, ^12^ ^]^

Introducing a “bridge unit” between the templates and the precursors is a promising approach to address the above problems, which has been demonstrated to be feasible in the fabrication of conventional mesoporous metal oxides.^[^ ^13^, ^14^ ^]^ Recently, the above strategy has been further applied to prepare the mesoporous crystalline materials.^[^ ^15^ ^]^ For example, Gu et al. creatively introduced Hofmeister salting‐in mediated anion (ClO_4_^−^) as a bridge to connect the organic ligands with the poly(ethylene oxide) corona of the triblock copolymer, and thus the precursor could be guided to grow and crystallize along the surfactant micelles, realizing the preparation of ordered mesoporous metal organic frameworks for the first time.^[^ ^16^ ^]^ Nevertheless, it is highly urgent to develop new routes for synthesis of the other mesoporous functional materials, especially for those that cannot be obtained by the well‐developed strategies.

Polyoxymetallates (POMs) are a new class of molecular clusters composed of transition metal ions (Mo, W, V, and Nb) and oxygen ligands.^[^ ^17^, ^18^ ^]^ The unique advantages including species diversity, high stability, and abundant oxygen‐containing acid coordination sites endow them with broad prospects in construction of the hierarchical mesoscopic superstructures.^[^ ^19^, ^20^ ^]^ Inspired by that, here we propose a facile strategy for POM‐assisted assembly to synthesize a series of superstructured mesoporous materials. The POMs act as bridge units to connect organic nitrogen–containing p‐phenylenediamine (PD) and surfactants through coordination and induced dipole–dipole interaction, respectively, to form complex micelles, and further co‐assemble to construct mesostructures. The resulting POM‐doped mesoporous polymer nanospheres showed the highly wrinkled lamellar superstructure with a uniform size of ≈208 nm, open radial mesoporous pores of 10‐20 nm, and high surface area of 67 m^2^ g^−1^. Further carbonization yields nitrogen‐doped hierarchical mesoporous carbon decorated uniformly with ultrafine *γ*‐Mo_2_N nanoparticles (less than 5 nm), which can effectively adsorb I_3_^−^ ions and alleviate the dissolution of polyiodides, thus remarkably improving the electrochemical performance of sodium–iodine (Na—I_2_) batteries. As expected, the carbonaceous composite is loaded with I_2_ as a cathode material, exhibiting excellent specific capacity (235 mA h g^−1^ at 0.5 A g^−1^), rate capability, and cycle stability.

## 2 Results and Discussion

**Figure 1:**
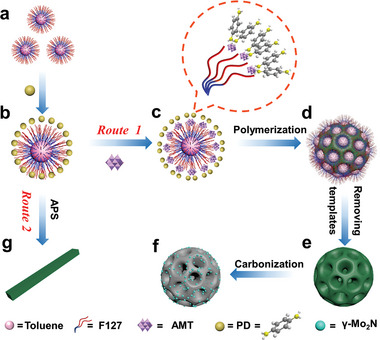
Route 1: Schematic illustration of introducing POMs (e.g., AMT) as bridge units to construct Mo‐mPPD and *γ*‐Mo_2_N/mNC. Route 2: Schematic illustration of the direct polymerization of monomers to generate the corresponding bulk partially crystalline polymer PPD, accompanied by the detachment of micelles.

**Figure 2:**
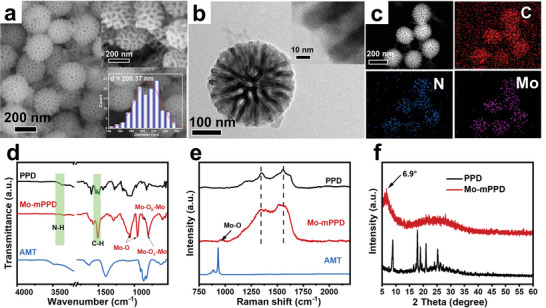
The structural characterization of mesoporous Mo‐mPPD. a) SEM image, b) TEM and HRTEM (inset) images, and c) EDX elemental mapping images of Mo‐mPPD sample. d) FT‐IR spectra and e) Raman spectra of PPD, AMT, and Mo‐mPPD samples. f) XRD patterns of PPD and Mo‐mPPD samples.

Route 1 of **Figure** **1** illustrates the bottom‐up preparation of mesoporous hybrid polymer materials by the POM‐assisted soft‐template co‐assembly (PASC) method. Commercial surfactant polyethylene oxide–polypropylene oxide–polyethylene oxide (EO_106_PO_70_EO_106_, F127) and small molecule PD were used as soft templates and organic precursors, respectively. Ammonium molybdate tetrahydrate (NH_4_)_6_Mo_7_O_24_·4H_2_O, AMT) containing homo‐polyacids (Figure S1, Supporting Information) was introduced as an “anionic bridge” to guide the assembly between F127 and PD. Ammonium persulfate was then added to trigger the polymerization of PD. Finally, the reaction solution was collected, and the template was further removed to obtain the product, which was named Mo‐mPPD (Figure S2, Supporting Information). The scanning electron microscope (SEM) image of Mo‐mPPD (**Figure** **2**a) reveals the well‐defined mesoporous nanosphere morphology with a uniform size of about 208 nm (based on the count of 130 particles). The pore wall is composed of continuous interwoven nanosheets with a thickness of about 10 nm, and the nanosheets are highly connected and appear as divergent dendrites, forming a large‐aperture open mesoscale structure. Transmission electron microscope (TEM) images (Figure 2b) further confirm the highly open radial channels arranged radially from the center to the surface, with pore size distribution of 10–20 nm and wall thickness around 10 nm. The energy dispersive X‐ray (EDX) elemental mapping images (Figure 2c) present a homogeneous distribution of the characteristic elements C, N, and Mo in the framework.

The nitrogen adsorption–desorption isotherm of Mo‐mPPD shows a typical type IV curve (Figure S3, Supporting Information), and the obvious capillary condensation hysteresis loop in the range of 0.8 < P/P_0_ < 1 indicates the existence of large mesopores. The pore size distribution curve presents a broad peak in the range of 10–20 nm by using Barrett–Joyner–Halenda (BJH) method, which is consistent with the results of SEM and TEM observations of open radial pores. The specific surface area is calculated about 67 m^2^ g^−1^. Fourier transform infrared (FT‐IR) spectroscopy of Mo‐mPPD (Figure 2d) displays two bands at 3420 and 3208 cm^−1^, which can be assigned to secondary amine stretching and terminal primary amine group vibrations, denoted N—H stretching. And the strong band at 1510 cm^−1^ is due to the stretching of the benzene ring backbone vibrations, respectively, which indicate the complete polymerization of PD monomer.^[^ ^21^ ^]^ Meanwhile, the characteristic peaks at 1062 (Mo—O), 958 (Mo—O), 874 (Mo—O_b_—Mo), and 805 (Mo—O_c_—Mo) evidence the hybridization of the inorganic phase.^[^ ^22^ ^]^ Furthermore, the disappearance of the characteristic signal around 2879 cm^−1^ (attributed to F127) prove the complete removal of the templating agent (Figure S4, Supporting Information).^[^ ^23^ ^]^ Raman spectra display their identical bands between 1400–1600 cm^−1^ of Mo‐mPPD and PPD, suggesting their similar polymer chain characteristics (Figure 2e). The characteristic peak at 978 cm^−1^ correspond to the Mo—O bond, further proving the hybridization of inorganic phase. The full spectrum of X‐ray photoelectron spectroscopy (XPS) shows four peaks at 230.8, 284.6, 400.1, and 530.3 eV, corresponding to Mo 3d, C 1s, N 1s, and O 1s, respectively (Figure S5, Supporting Information).^[^ ^24^ ^]^ Quantitative XPS analysis shows that the atom content of Mo and N elements in Mo‐mPPD hybrids is 3.05% and 19.5% (Table S1, Supporting Information), respectively. The Mo 3d spectrum can be fitted into 231.9 and 235.08 eV, which can be ascribed to Mo^6+^ and Mo^5+^, indicating the interaction between the inorganic phase and the organic phase.^[^ ^25^ ^]^

To gain insight into the role of POMs, we carried out a control experiment without adding POMs using a similar procedure, the resulting product is named PPD. SEM images (Figure S6, Supporting Information) of PPD display a long rod morphology with width of about 125 nm. The corresponding X‐ray diffraction (XRD) pattern (Figure 2f) shows the several sharp peaks between 2*θ* = 8–30°, indicating the presence of a crystalline phase in PPD.^[^ ^26^ ^]^ The amino groups of PD can form hydrogen bonds with the ether oxygen groups of F127,^[^ ^27^ ^]^ this can also be confirmed by the significant change in the zeta potential of the F127 dispersion after adding PD (Figure S7, Supporting Information). Ultraviolet‐visible (UV–vis) spectrum of PPD (Figure S8, Supporting Information) displays two peaks at 243 and 306 nm, belonging to different *π*–π* transition of benzenoid ring.^[^ ^28^ ^]^ Unfortunately, the *π*–*π* stacking of PD to form crystals may be stronger than the hydrogen bonding between PD and F127, resulting in micelles being expelled, which cannot guide the formation of mesoporous structures, and only crystalline nanorods can be obtained (Figure 1, Route 2). After the introduction of AMT containing a large number of negatively charged oxygen‐containing clusters, a strong coordination bond will be formed with positively charged PD, which is further confirmed by change of UV–vis spectroscopy (Figure S9, Supporting Information) and the color change in the mixed solution (Figure S2, Supporting Information).^[^ ^25^ ^]^ Meanwhile, the ether oxygen groups in the outer corona of F127 can form induced dipole–dipole interactions with the hydronium ions of AMT (Figure S10, Supporting Information).^[^ ^29^ ^]^ Therefore, AMT serves as a bifunctional bridge connecting micelles and monomers, which can avoid the orientation accumulation of monomers during the polymerization process, and also can introduce soft templates into the entire assembly process to construct mesoporous structures (Figure 1, Route 1). All the characteristic peaks of the partial crystalline polymer in the XRD pattern (Figure 2f) disappear after adding AMT, indicating that the AMT plays a key role in the formation of Mo‐mPPD. The new peak at 6.9° may represent the intercalation structure of the hybrid product. In addition, POMs can also introduce metal anion clusters into soft polymer chains in situ, realizing host–guest complementary combination and synergistic effect at the molecular scale. Accordingly, a variety of flexible hybrid frameworks with controllable components are obtained from bottom to top. Impressively, by varying POMs types with similar property such as H_4_[Si(W_3_O_10_)_4_]·*x*H_2_O, Na_3_O_40_·PW_12_·*x*H_2_O, and H_3_PO_4_·12MoO_3_, mesoporous hybrid nanospheres with different compositions were also synthesized. All of which showed similar mesoporous nanosphere morphologies (Figure S11, Supporting Information), which further demonstrates the key role of POMs as bridging molecules.

The component concentrations of precursors and monomers are optimal in the experiments at present. In addition to the above types of POMs, the micelle system also affects the morphology and nanostructure of the material. To understand the formation mechanism of Mo‐PPD, the correlation between mesoporous structure and the dosage of F127, toluene, and AMT during the assembly process was systematically studied. More specifically, in the case of a fixed amount of PD, as the molar ratio of AMT increases, the morphologies of product gradually changed from irregular particles to hollow mesoporous spheres, dendritic mesoporous wrinkled spheres, and larger‐size gathering ball (Figure S12, Supporting Information). Combined with the above results of PPD fiber rods (Figure S6, Supporting Information), this comparison further validates the previously mentioned role of AMT as an anion bridge during self‐assembly process. Further increase of AMT will form irregularly granular organic‐polyoxometalate co‐crystals instead of mesoporous architecture and spherical particles.^[^ ^30^ ^]^ This may be attributed to the fact that the coordination between the PD and AMT is much larger than that between AMT and F127 at this ratio, resulting in F127 being expelled during the polymerization growth. In additionas a soft template to guide the formation of mesopores, the F127 also acts as a surfactant to regulate the formation of spherical morphology. As shown in Figure S13, Supporting Information, the product of the control experiment without adding F127 presented an irregular particle piled up by disorganized fine fibers. Moreover, in the absence of toluene, only nonporous spherical particles were obtained (Figure S14, Supporting Information). As the amount of toluene in the micellar system increased, the nanospheres gradually changed from shallow‐pored golf balls to dendritic mesoporous spheres with larger pore diameters and thicker pore walls. Accordingly, the hydrophobic phase toluene acts as a swelling agent in our system, which enlarges the size of the micelles by changing the hydrophilic–hydrophobic ratio, and can also adjust the morphology and mesoporous structure of the final product, which is consistent with previous reports.^[^ ^7^, ^31^ ^]^

The above experiments indicated that this PASC strategy mainly relies on the addition of appropriate amounts of bridging molecules and the formation of stable complex micelles. Based on the above observations, a robust PASC strategy is proposed for the synthesis of a series of mesoporous organic‐polyoxometalate hybrid spherical nanoparticles. As shown in Figure 1a, the amphiphilic surfactant F127 first self‐assembled in a water/ethanol mixed solution to form spherical micelles with hydrophobic PPO as the core and hydrophilic PEO as the shell. The toluene will enter the inner core of the micelles through Van der Waals' force to expand micelles size and stabilize the micelles.^[^ ^32^ ^]^ After adding PD monomers, the corona of F127 consisting of PEO chains form hydrogen bonds with PD (Figure 1b). The subsequently added POMs bind the micelles through induced dipole–dipole interactions between hydronium ions and ether oxygen groups of the micelles, and connect the organic precursors through coordination to form F127‐POM‐PD subunit, preventing excessive accumulation between PD monomers (Figure 1c). The composite micelles are further co‐assembled into low‐energy and close‐packed supramolecular assemblies. After adding an oxidizing agent, the monomers wrap the micelles to in situ cross‐link and polymerize to form a polymer network, and the metal anion clusters are embedded in the polymer chain (Figure 1d). Due to the isotropic growth of the cross‐linking process in solution, uniform nanospheres with well‐defined mesoscale pores can be obtained after the final removal of the soft template (Figure 1e).^[^ ^33^ ^]^

**Figure 3:**
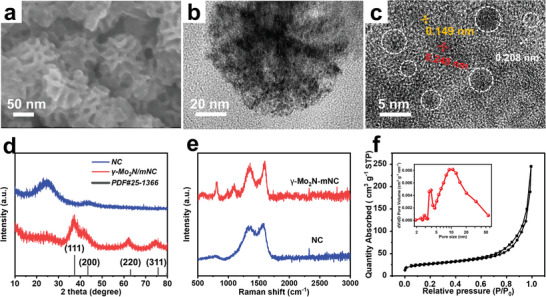
The structural characterization of mesoporous *γ*‐Mo_2_N/mNC sample. a) SEM image, b) TEM image, and c) HRTEM of *γ*‐Mo_2_N/mNC sample, d) XRD patterns and e) Raman spectra of NC and *γ*‐Mo_2_N/mNC samples, and g) N_2_ adsorption–desorption isotherms and corresponding pore size distribution (inset) of *γ*‐Mo_2_N/mNC sample.

After annealing Mo‐mPPD sample in an inert atmosphere, the template can be completely removed and mesoporous N‐doped carbon nanospheres are finally prepared (Figure 1f). This process promotes the reaction between molybdenum‐containing components and N‐doped carbon components, deriving hierarchical porous carbon superstructure with uniform dispersion of ultrafine molybdenum nitride nanoparticles (denoted as *γ*‐Mo_2_N/mNC). Nitrogen‐doped carbon (NC) was also synthesized under the same conditions for comprehensive analyst of characterizations. The SEM image (**Figure** **3**a) represents that the mesoporous morphology of *γ*‐Mo_2_N/mNC after high temperature carbonization is well retained and the average particle size is 109 nm. EDX images (Figure S15, Supporting Information) depict that C, N, O, and Mo elements are uniformly distributed. The TEM image (Figure 3b) shows the uniform dispersion of molybdenum nitride with a particle size of about 2–5 nm, which may be ascribed to the mesoporous structure and in situ polymerization hindering the aggregation and growth of metal species. The HRTEM image (Figure 3c) shows that the lattice fringes spacing of the Mo‐based particles is 0.240, 0.208, and 0.149 nm, corresponding to the (111), (200), and (220) planes of *γ*‐Mo_2_N (PDF#25‐1366), respectively.^[^ ^34^ ^]^ As can be seen from Figure 3d, the characteristic peaks of *γ*‐Mo_2_N/mNC at 2*θ* = 37.0°, 41.6°, 61.8°, and 74.5° in the XRD pattern correspond to (111), (200), (220), and (311) crystal facets, respectively.^[^ ^35^ ^]^ Obviously, (111) is the dominant exposed crystal plane, and the corresponding weaker peak intensity may be caused by the small grain size of *γ*‐Mo_2_N, which is consistent with the results observed by TEM. Besides, similar to NC, the broad diffraction peak at 26° belongs to partially graphitic carbon, which is further supported by the Raman spectrum in Figure 3e. The spectrum of *γ*‐Mo_2_N/mNC in Figure 3e shows two broad peaks at 1345 and 1583 cm^−1^, which belong to disordered sp^3^ carbon (D band) and graphitic sp^2^ carbon (G band), respectively, and the spectrum displays sharp peaks and fingerprint bands characteristic of *γ*‐Mo_2_N at low‐band region, certifying that the *γ*‐Mo_2_N phase is homogeneously doped in carbon matrix.^[^ ^36^ ^]^

The nitrogen adsorption–desorption isotherm of *γ*‐Mo_2_N/mNC shows a typical type IV curve (Figure 3f), and the pore size distribution curve indicates its hierarchical porous nature, where the 2–3 nm pores may be left by the regrowth of metal species, and the size of mesopores shrinks to 12 nm due to the heat treatment (Figure 3f, inset). The specific surface area of *γ*‐Mo_2_N/mNC calculated by the BJH method is 92 m^2^ g^−1^. The XPS analysis of *γ*‐Mo_2_N/mNC can provide a deeper understanding of their composition and valence state. The full spectrum of *γ*‐Mo_2_N/mNC shows distinct peaks corresponding to C, N, O, and Mo element (Figure S16a, Supporting Information), and the contents of Mo element increases after carbonization (Table S1, Supporting Information). The deconvoluted C 1s spectrum in Figure S16b, Supporting Information, reveals four peaks located at 284.5, 285.3, 286.3, and 289.2 eV belong to the C—C, C—O, and C=C bonds, respectively.^[^ ^25^ ^]^ In the high‐resolution N 1s spectrum (Figure S16c, Supporting Information), the peak at 397.6 eV confirms that the Mo element interacts with the N element in the framework to form Mo—N bonds during carbonization process, and the remaining four peaks at 395.3, 398.7, 400.2, and 402.0 eV are assigned Mo 3p (overlap), pyridinic N, pyrrolic N, and graphitic N, respectively, where pyridinic N is the predominant N species. The high‐resolution Mo 3d spectrum (Figure S16d, Supporting Information) can be deconvoluted into six peaks.^[^ ^37^ ^]^ The peaks at 229.4 and 232.5 eV are attributed to the Mo^4+^ chemical state, and the peaks at 236.0 and 233.0 eV are ascribed to the Mo^6+^ chemical state. Notably, the peak at 228.8 eV represents the formation of the *γ*‐Mo_2_N phase. The remaining peak can be assigned to Mo^*ζ*+^ in other chemical valence states on the material surface, which is ascribed to the inevitable surface oxidation.^[^ ^38^ ^]^

Na—I_2_ batteries are promising candidates for energy storage devices due to their high theoretical capacity (211 mA h g^−1^), distinguished reversibility, and environmental friendliness.^[^ ^39^, ^40^ ^]^ However, the poor electronic conductivity of I_2_ (10^−6^ S cm^−1^) and its high solubility in organic electrolytes lead to severe capacity fading and low Coulombic efficiency (CE) of Na—I_2_ batteries.^[^ ^41^ ^]^ The ideal I_2_ host for Na—I_2_ batteries cathode is characterized by high I_2_ loading, great electronic conductivity, and electrochemical activity, which promote the adsorption and accelerate the conversion process of iodine trianions (I_3_^−^) during the charge and discharge process. N heteroatoms doped porous carbon shows catalytic activity for I_2_ redox reactions.^[^ ^42^ ^]^ Additionally, metal nitrides have been reported to exhibit great chemisorption and catalytic activity toward polysulfides in lithium–sulfur batteries.^[^ ^43^ ^]^ Given that metal–I_2_ batteries and metal–sulfur batteries possess the similar slow precipitation–dissolution kinetics during redox chemical process, it can be inferred that metal nitride has the prospect and possibility of being applied to metal–iodine batteries.^[^ ^44^ ^]^ Recently, mesoporous structures have been reported to be used in the construction of advanced metal–iodide battery cathodes with excellent performance, which can shorten the diffusion pathways of ions and electrons, and confine the polyiodide/I^−^ conversion reaction inside the mesopores.^[^ ^45^ ^]^ Considering the advantages of *γ*‐Mo_2_N/mNC multi‐active components, high specific surface area, and unique mesoporous structure, it is expected to improve the electrochemical performance of metal–iodine batteries. As a proof of concept, we used the *γ*‐Mo_2_N/mNC as the host material of I_2_ and further applied in Na—I_2_ batteries. Using the sublimation diffusion method, 25 wt% I_2_ was loaded into *γ*‐Mo_2_N/mNC to obtain the I_2_@*γ*‐Mo_2_N/mNC composites. The SEM image demonstrates that the morphology and mesoporous structure of the hybrid remain unchanged after loading I_2_, and EDX images (Figure S17, Supporting Information) prove that I_2_ is uniformly loaded on *γ*‐Mo_2_N/mNC. The XRD pattern (Figure S18, Supporting Information) further verified that no characteristic peak of I_2_ appeared after impregnation, indicating no large block aggregation of I_2_. To compare the effect of mesopores on battery performance, non‐porous *γ*‐Mo_2_N/CN was synthesized as a control sample, and then the same operation was performed to generate a comparison sample I_2_@*γ*‐Mo_2_N/NC. The XRD pattern (Figure S19, Supporting Information) and EDX images (Figure S20, Supporting Information) suggest their similar properties of I_2_ distribution.

**Figure 4:**
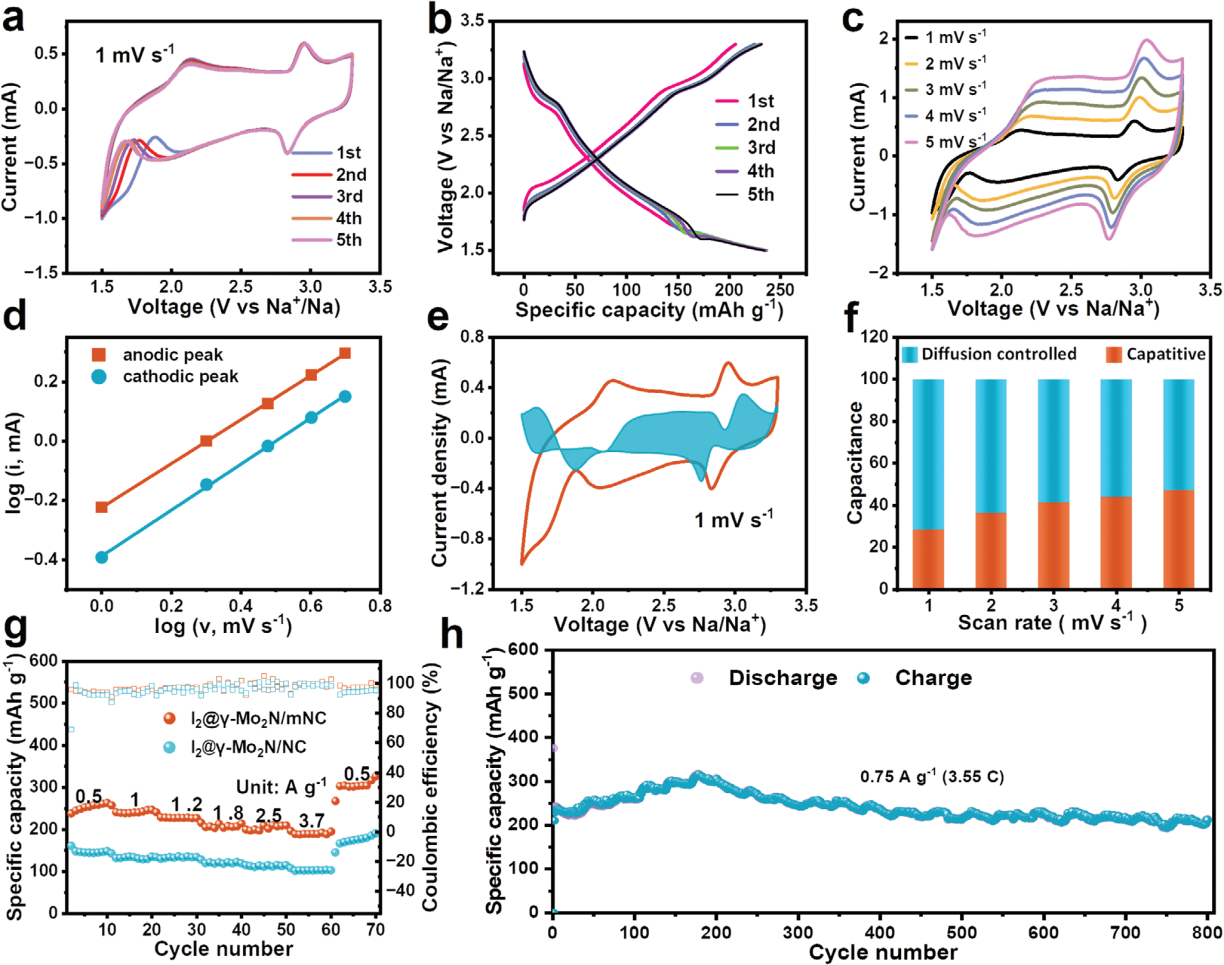
Electrochemical performance of Na—I_2_ battery cathodes. a) CV curves of I_2_@*γ*‐Mo_2_N/mNC cathode at a scan rate of 1 mV s^−1^. b) GCD curves of I_2_@*γ*‐Mo_2_N/mNC electrode at a current density of 0.5 A g^−1^. c) CV curves of I_2_@*γ*‐Mo_2_N/mNC cathode at different scan rates. d,e) Illustration of the calculated capacitive and diffusion‐controlled contributions of I_2_@*γ*‐Mo_2_N/mNC electrode at 1 mV s^−1^. f) Calculated capacitive and diffusion‐controlled contribution of I_2_@*γ*‐Mo_2_N/mNC electrode at different scan rates. g) Rate capability of I_2_@*γ*‐Mo_2_N/mNC and I_2_@*γ*‐Mo_2_N/NC electrodes.h) Cycling performance of I_2_@γ‐Mo_2_N/mNC.

To study the redox activity of I_2_@*γ*‐Mo_2_N/mNC cathode, sodium foil was used as anode, 1M NaClO_4_ in organic mixed solvent was used as electrolyte, and glass fiber was used as the separator, to assemble a Na—I_2_ coin‐type battery. As the references, pure *γ*‐Mo_2_N/mNC without loading I_2_ and I_2_@*γ*‐Mo_2_N/NC cathodes were also tested under the same conditions. Cyclic voltammetry (CV) tests were performed to investigate the redox reactions of different electrodes during the charging and discharging process at different scan rates. **Figure** **4**a shows the CV curves of I_2_@*γ*‐Mo_2_N/mNC cathode for the first five cycles at a scan rate of 1 mV s^−1^. As shown in Figure S21, Supporting Information, compared to the deformed rectangle of *γ*‐Mo_2_N/mNC cathode without obvious redox peaks, I_2_@*γ*‐Mo_2_N/mNC cathode displays a pair of redox sharp peaks at ≈2.84 and 2.95 V. The redox peaks overlap well in the first five cycles without obvious shift, attributing to the great NaI_3_/NaI reversible conversion. Compared with the I_2_@*γ*‐Mo_2_N/NC electrode, the humps at about 1.75 and 2.14 V may be ascribed to the I_2_ confinement effect caused by mesopores. As the charge–discharge process proceeds, the electrolyte gradually diffuses into the interior of the mesopores, causing the hump to shift. In contrast, the polarization voltage of I_2_@*γ*‐Mo_2_N/mNC cathode is much smaller, the sharper peak and the larger closed area indicates the faster reaction kinetics and higher capacity, which may be attributed to the high accessible specific surface area and abundant exposed active sites brought about by the unique mesoporous structure. Additionally, Figure 4b corresponds to the discharge–charge curves of the first five cycles for the I_2_@*γ*‐Mo_2_N/mNC electrode at a current density of 0.5 A g^−1^, which presents a pair of symmetrical voltage plateaus at 2.7–2.9 V, matching well with the CV curves. In addition, the CE is close to 100%, demonstrating its excellent reversibility and high I_2_ utilization.

Figure 4c presents the CV curves of the I_2_@*γ*‐Mo_2_N/mNC electrode at various scan rates in the range of 1–5 mV s^−1^. The scan rate was expanded to five times, I_2_@*γ*‐Mo_2_N/mNC electrode still exhibited a small potential shift (64 mV), and the polarization voltage was always below 250 mV (2.77/3.02 V), proving its stable redox chemistry. Further calculations were carried out to evaluate the electrochemical contribution of I_2_@*γ*‐Mo_2_N/mNC electrode. The degree of capacitive effect can be qualitatively analyzed according to the relationship between the measured CV current (*i*) and the scan rate (*v*) by the equation: *i* = *av^b^*, where *a* is an adjustable constant, and the value of *b* is determined by the slope of the log(*i*) versus log(*v*) plot.^[^ ^46^ ^]^ As shown in Figure 4d, the *b* values of the cathodic scanning and anodic scanning processes are ≈0.77 and ≈0.74 respectively, implying that the I_2_@*γ*‐Mo_2_N/mNC electrode possesses capacitance‐controlled and diffusion‐controlled storage kinetics. According to the formula *i = k*_1_ *v + k*_2_ *v* ^1/2^, the total current under each voltage on the CV curve can be divided into diffusion‐controlled current and capacitive current, where *i* and *v* represent the total current and scan rate, respectively, *k*_1_ *v* and *k*_2_ *v* ^1^ *^/^* ^2^ belong to the current contributions from the capacitive effect and the diffusion‐controlled process, respectively.^[^ ^47^ ^]^ As shown in Figure 4e,f, when the scan rate is increased from 1 to 5 mV s^−1^, the contribution of capacitance increases from 28% to 47%. Usually, the I_2_/NaI redox reaction is mainly dominated by the diffusion‐controlled charge storage process, so the capacitive contribution is attributed to the intrinsic pseudocapacitance of *γ*‐Mo_2_N/mNC electrode, which can improve its rate performance.

Subsequently, the rate capability and cycle performance of I_2_@*γ*‐Mo_2_N/mNC and I_2_@*γ*‐Mo_2_N/NC electrodes were investigated by galvanostatic charge–discharge (GCD) test. As shown in Figure 4g, the specific capacities of I_2_@*γ*‐Mo_2_N/mNC cathode are 254, 240, 228, 204, 202, 189, and 137 mAh g^−1^ at current densities of 0.5, 1, 1.2, 1.8, 2.5, and 3.7 A g^−1^, respectively, which are significantly higher than I_2_@*γ*‐Mo_2_N/NC electrode. The specific capacity at low current densities is higher than the theoretical capacity of I_2_ (211 mAh g^−1^), which may be the capacity contributed by *γ*‐Mo_2_N/mNC electrode to Na^+^ and the storage of surface pseudocapacitance (Figure S22, Supporting Information). The charge–discharge curves in Figure S23, Supporting Information, reveal their CE remains above 90% at low discharge currents, and a discharge plateau at around 2.8 V can still be distinguished at high discharge current densities, which demonstrates its excellent redox reversibility and high I_2_ utilization. After repeated charge and discharge at different current densities, when the current density returns to 0.5 A g^−1^, the corresponding capacity increases to 303 mAh g^−1^, which may be due to the gradual diffusion of the electrolyte into the mesopores and activates the I_2_ anchored inside the mesopores to participate in the redox reaction. Notably, the rate performance of I_2_@*γ*‐Mo_2_N/mNC electrode exceeds that of previously reported cathode materials, including carbon, conducting polymer, and MOF materials (Figure S24, Supporting Information). This can be attributed to the high specific surface area of the nanospheres leading to more electrolyte‐accessible active sites, and the open mesopores can shorten the transport path and facilitate ion diffusion. In addition, the dispersed *γ*‐Mo_2_N nanoparticles can accelerate the catalytic conversion of I_2_, leading to significantly improved discharge capacity and rate performance (Figure S25, Supporting Information). Subsequently, the cycling performance of I_2_@*γ*‐Mo_2_N/mNC and I_2_@*γ*‐Mo_2_N/NC electrode was studied. Figure S26, Supporting Information, shows that I_2_@*γ*‐Mo_2_N/mNC‐base battery can work stably for 200 cycles with a higher specific capacity of 300 mAh g^−1^at a current density of 0.75 A g^−1^, higher than the non‐porous sample (192 mAh g^−1^). In addition to the gap in discharge capacity, both of them show highcapacity retention and CE value, which implies that compared with the mesoporous structure, the electrode composition plays a dominant role in improving the cycle performance of Na—I_2_ batteries. As can be seen in Figure 4h, the discharge capacity of the I_2_@*γ*‐Mo_2_N/mNC electrode rises slightly, and then slowly declines. It still shows a discharge capacity of 213.5 mAh g^−1^ at 800 cycles, showing good cycle stability.

**Figure 5:**
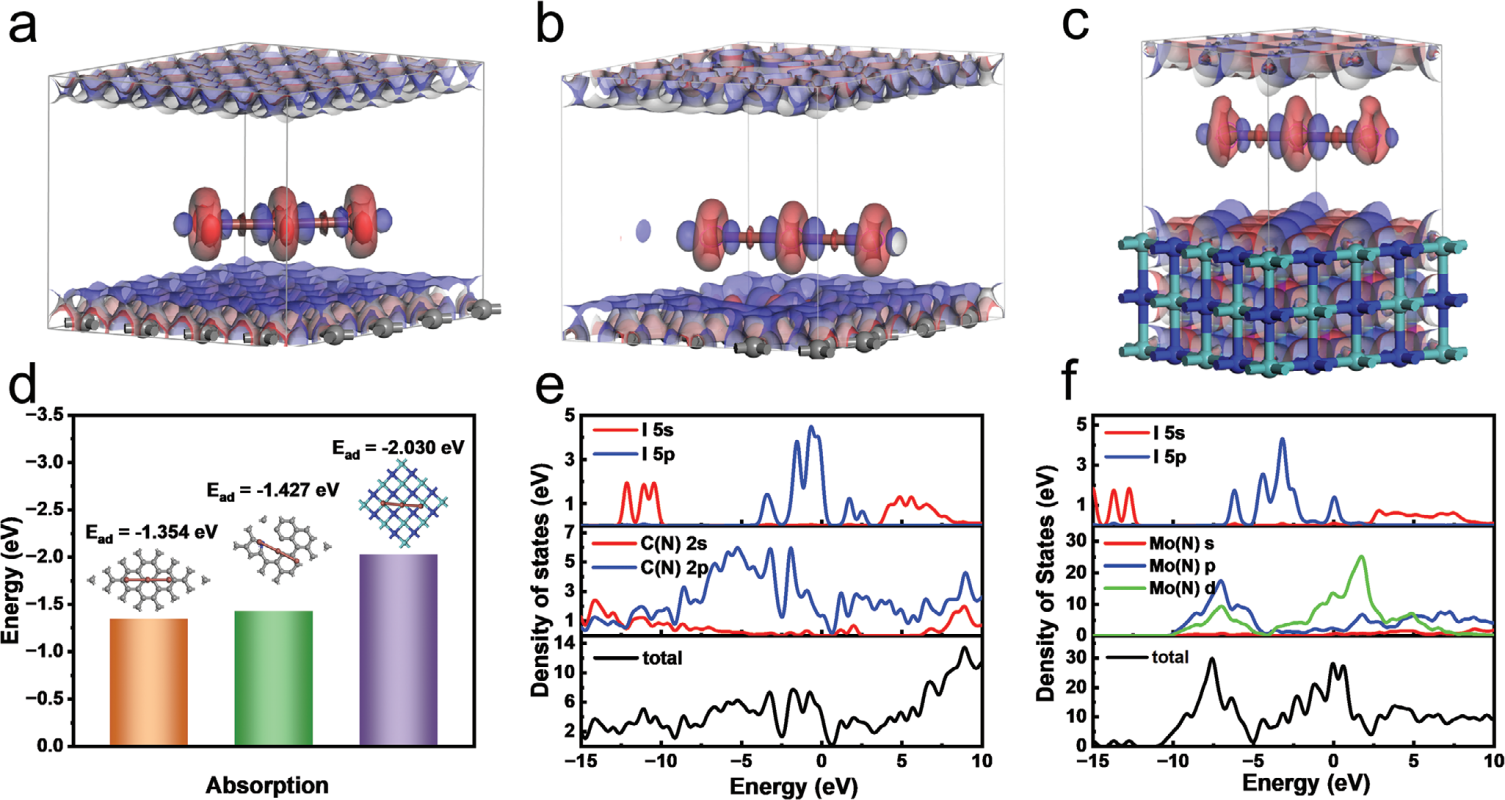
Charge density difference of the optimized configurations of a) graphene, b) NC, and c) *γ*‐Mo_2_N after I_3_^−^ adsorption (grey: C, blue: N, light blue: Mo, red: I). d) Binding energies between I_3_^−^ and graphene, NC, and *γ*‐Mo_2_N. e,f) Density of states analysis for NC—I_3_^−^ and *γ*‐Mo_2_N—I_3_^−^, respectively.

To further explore above electrochemical differences, a thermodynamic analysis of the entire reaction was performed using density functional theory (DFT) calculations and shown in **Figure** **5**. Adsorption is the first step in the catalytic reaction, and the calculated binding energy of adsorption can be used to explain the affinity of iodide ions. Figure 5d shows the calculation results of the binding energy of I_3_^−^ on graphene, NC, and *γ*‐Mo_2_N as models. The binding energy of *γ*‐Mo_2_N to I_3_^−^ is the −2.030 eV, lower than that of NC (−1.427 eV) and graphene plane (−1.354 eV). Compared with graphene, the increased polarity of nitrogen‐doped sites will enhance the interaction with I_2_, which facilitates the nucleation of I_2_ as it lowers the surface tension of the carbon substrate, and lowers activation energy and overpotential of the process. Meanwhile, the *γ*‐Mo_2_N phase reveals outstanding anchoring performance to I_3_^−^ and can significantly suppress the shuttling effect. In order to understand the root cause of the difference in the adsorption of I_3_^−^ ions on graphene monolayers, pyridinic nitrogen, and *γ*‐Mo_2_N, we calculated their electron density difference after adsorbing I_3_^−^. The electron accumulation and electron depletion are corresponded to the blue region and red region in Figure 5a–c, respectively. As can be seen from Figure 5a, the electron distribution on the graphene monolayer shows no change, while the carbon material appears obvious charge transfer after introducing pyridinic N. Consequently, the active center of pyridinic N‐doped carbon is the pyridinic N atom instead of the carbon atom, which is mainly attributed to the lone pair of electrons in the pyridinic N atom, and the charge redistribution occurs between the pyridinic nitrogen carbon and I_3_^−^ after the interaction.^[^ ^48^ ^]^ Combined with the more obvious charge rearrangement after *γ*‐Mo_2_N adsorbed I_3_^−^, reflecting a strong chemical ability of NC and polar *γ*‐Mo_2_N for anchoring I_3_^−^, which can effectively suppress the excessive dissolution of NaI_3_. Simultaneously, we calculated density of states (DOS) before and after the adsorbing I_3_^−^ on NC and *γ*‐Mo_2_N, as shown in Figure 5e,f. DOS calculations show that the interaction between the two models and I_3_^−^ is mainly due to the overlap the p orbital of I_3_^−^ with the p orbital of C(N), and the d orbital of Mo(N), respectively. In contrast, the DOS of I_3_^−^ adsorbed on Mo(N) exhibited a wider energy band distribution and shifted to lower energy levels, proving that the *γ*‐Mo_2_N and I_3_^−^ are easier to bond. The above results indicate the strong hybridization of I atoms between the N atoms and the orbitals of *γ*‐Mo_2_N. Consequently, the carbon materials doped with N and *γ*‐Mo_2_N can effectively stabilize I_3_^−^, endowing I_2_@*γ*‐Mo_2_N/mNC with a high specific capacity and good cycling ability.

## 3 Conclusions

We propose a new strategy for the controllable preparation of a series of mesoporous polymer nanospheres with different compositions and morphologies by introducing POMs as bifunctional bridging units. Among them, the AMT‐bridged co‐assembly route can obtain Mo‐mPPD with highly wrinkled walls and conical aperture radially outward from the center. After carbonization, corresponding nitrogen‐doped hierarchical porous carbon hybrids with uniformly dispersed ultrafine molybdenum nitride nanoparticles is obtained. Further loading I_2_ and acting as cathode material for Na—I_2_ battery, the I_2_@*γ*‐Mo_2_N/mNC electrode exhibits high discharge capacity, excellent rate capability, and cycle stability. DFT proves that the adsorption capacity of nitrogen‐doped carbon and *γ*‐Mo_2_N on polyiodide can alleviate the shuttling effect in organic electrolytes. This work can provide a new insight for constructing functional mesoporous materials, and also expand their promising applications.

## Conflict of Interest

The authors declare no conflict of interest.

## Supporting information

Supporting Information

## Data Availability

The data that support the findings of this study are available in the supplementary material of this article.

## References

[1] R. Zhang , Z. Liu , T. N. Gao , L. Zhang , Y. Zheng , J. Zhang , L. Zhang , Z. A. Qiao , Angew. Chem., Int. Ed. 2021, 60, 24299. 10.1002/anie.202111239 34498361

[2] J. Ma , Y. Li , J. Li , X. Yang , Y. Ren , A. A. Alghamdi , G. Song , K. Yuan , Y. Deng , Adv. Funct. Mater. 2022, 32, 2107439.

[3] Y. Zheng , L. Wang , H. Liu , J. Yang , R. Zhang , L. Zhang , Z.‐A. Qiao , Angew. Chem., Int. Ed. 2022, 61, e202209038. 10.1002/anie.202209038 35864559

[4] F. Wei , T. Wang , X. Jiang , Y. Ai , A. Cui , J. Cui , J. Fu , J. Cheng , L. Lei , Y. Hou , Adv. Funct. Mater. 2020, 30, 2002092.

[5] J. Fang , L. Zhang , J. Li , L. Lu , C. Ma , S. Cheng , Z. Li , Q. Xiong , H. You , Nat. Commun. 2018, 9, 521. 29410431 10.1038/s41467-018-02930-9 PMC5802826

[6] M. Chen , H. Xuan , X. Zheng , J. Liu , X. Dong , F. J. E. A. Xi , Adv. Sci. 2017, 238, 269.

[7] L. Peng , H. Peng , C. Hung , D. Guo , L. Duan , B. Ma , L. Liu , W. Li , D. Zhao , Chem 2021, 7, 1020.

[8] C. Liang , S. Dai , J. Am. Chem. Soc. 2006, 128, 5316. 16620083 10.1021/ja060242k

[9] D. Gu , F. Schüth , Chem. Soc. Rev. 2014, 43, 313. 23942521 10.1039/c3cs60155b

[10] K. Li , J. Yang , J. Gu , Acc. Chem. Res. 2022, 55, 2235. 35904471 10.1021/acs.accounts.2c00262

[11] Y. Wang , Y. Liu , H. Li , X. Guan , M. Xue , Y. Yan , V. Valtchev , S. Qiu , Q. Fang , J. Am. Chem. Soc. 2020, 142, 3736. 32050755 10.1021/jacs.0c00560

[12] Q. Yin , Y Li , L. Li , J. Lü , T. Liu , R. Cao , ACS Appl. Mater. Interfaces 2019, 11, 17823. 31009575 10.1021/acsami.9b03696

[13] C. Jo , J. Hwang , W. G. Lim , J. Lim , K. Hur , J. Lee , Adv. Mater. 2018, 30, 1703829. 10.1002/adma.201703829 29271508

[14] W. Luo , Y. Li , J. Dong , J. Wei , J. Xu , Y. Deng , D. Zhao , Angew. Chem., Int. Ed. 2013, 125, 10699.

[15] K. Li , Y. Zhao , J. Yang , J. Gu , Nat. Commun. 2022, 13, 1879. 35388007 10.1038/s41467-022-29535-7 PMC8986779

[16] K. Li , J. Yang , R. Huang , S. Lin , J. Gu , Angew. Chem., Int. Ed. 2020, 132, 14228.

[17] Y. Song , R. Tsunashima , Chem. Soc. Rev. 2012, 41, 7384. 22850732 10.1039/c2cs35143a

[18] H. Zhang , W. Zhao , H. Li , Q. Zhuang , Z. Sun , D. Cui , X. Chen , A. Guo , X. Ji , S. An , Polyoxometalates 2022, 1, 9140011.

[19] Q. Liu , Q. Zhang , W. Shi , H. Hu , J. Zhuang , X. Wang , Nat. Chem. 2022, 14, 433. 35145248 10.1038/s41557-022-00889-1

[20] J. Li , Z. Chen , M. Zhou , J. Jing , W. Li , Y. Wang , L. Wu , L. Wang , Y. Wang , M. Lee , Angew. Chem., Int. Ed. 2016, 55, 2592. 10.1002/anie.201511276 26766581

[21] Z. Liu , H. Zhou , Z. Huang , W. Wang , F. Zeng , Y. Kuang , J. Mater. Chem. 2013, 1, 3454.

[22] C. Chen , S. Mao , C. Tan , Z. Wang , Y. Ge , Q. Ma , X. Zhang , G. Qi , J. Xu , Z. Fan , Angew. Chem., Int. Ed. 2021, 60, 15556. 10.1002/anie.202104028 33942452

[23] Q. Li , X. Xu , J. Guo , J. P. Hill , H. Xu , L. Xiang , C. Li , Y. Yamauchi , Y. Mai , Angew. Chem., Int. Ed. 2021, 133, 26732. 10.1002/anie.202111823 34748252

[24] M. Y. Zhang , Y. Song , D. Yang , Z. Qin , D. Guo , L. J. Bian , X. G. Sang , X. Sun , X. X. Liu , Adv. Funct. Mater. 2021, 31, 2006203.

[25] Y. Guo , J. Tang , J. Henzie , B. Jiang , H. Qian , Z. Wang , H. Tan , Y. Bando , Y. Yamauchi , Mater. Horiz. 2017, 4, 1171.

[26] S. Yang , F. Liao , Synth. Met. 2012, 162, 1343.

[27] C. Li , H. Peng , J. Cai , L. Li , J. Zhang , Y. Mai , Adv. Mater. 2021, 33, 2102930. 10.1002/adma.202102930 34170570

[28] J. Wang , J. Jiang , B. Hu , S. Yu , Adv. Funct. Mater. 2008, 18, 1105.

[29] J. Ettedgui , R. Neumann , J. Am. Chem. Soc. 2009, 131, 4. 19128167 10.1021/ja808523n

[30] S. Li , Z. Zhao , T. Ma , P. Pachfule , A. Thomas , Angew. Chem., Int. Ed. 2022, 61, e202112298. 10.1002/anie.202112298 PMC9300107 34709716

[31] R. Wang , K. Lan , R. Lin , X. Jing , C.‐T. Hung , X. Zhang , L. Liu , Y. Yang , G. Chen , X. Liu , C. Fan , A. M. El‐Toni , A. Khan , Y. Tang , D. Zhao , ACS Nano 2021, 15, 7713. 33821624 10.1021/acsnano.1c01367

[32] L. Peng , C. Hung , S. Wang , X. Zhang , X. Zhu , Z. Zhao , C. Wang , Y. Tang , W. Li , D. Zhao , J. Am. Chem. Soc. 2019, 141, 7073. 30964289 10.1021/jacs.9b02091

[33] Y. Fang , D. Gu , Y. Zou , Z. Wu , F. Li , R. Che , Y. Deng , B. Tu , D. Zhao , Angew. Chem., Int. Ed. 2010, 122, 8159. 10.1002/anie.201002849 20839199

[34] Y. Huang , W. Zhou , W. Kong , L. Chen , X. Lu , H. Cai , Y. Yuan , L. Zhao , Y. Jiang , H. Li , Adv. Sci. 2022, 9, 2204949. 10.1002/advs.202204949 PMC9799021 36285692

[35] S. A. Pandey , C. Zhang , D. H. Ibrahim , E. A. Goldfine , J. K. Wenderott , R. Dos Reis , R. L. Paul , I. Spanopoulos , M. Kanatzidis , M. J. Bedzyk , Chem. Mater. 2021, 33, 6671.

[36] Z. Yu , X. An , I. Kurnia , A. Yoshida , Y. Yang , X. Hao , A. Abudula , Y. Fang , G. Guan , ACS Catal. 2020, 10, 5353.

[37] R. Li , X. Zhou , H. Shen , M. Yang , C. Li , ACS Nano 2019, 13, 10049. 31433615 10.1021/acsnano.9b02231

[38] F. Ma , K. Srinivas , X. Zhang , Z. Zhang , Y. Wu , D. Liu , W. Zhang , Q. Wu , Y. Chen , Adv. Funct. Mater. 2022, 32, 2206113.

[39] F. Wang , Z. Liu , C. Yang , H. Zhong , G. Nam , P. Zhang , R. Dong , Y. Wu , J. Cho , J. Zhang , Adv. Mater. 2020, 32, 1905361. 10.1002/adma.201905361 31815328

[40] C. Guo , B. Han , W. Sun , Y. Cao , Y. Zhang , Y. Wang , Angew. Chem., Int. Ed. 2022, 134, e202213276. 10.1002/anie.202213276 36196009

[41] L. Xiang , S. Yuan , F. Wang , Z. Xu , X. Li , F. Tian , L. Wu , W. Yu , Y. Mai , J. Am. Chem. Soc. 2022, 144, 15497. 35979963 10.1021/jacs.2c02881

[42] T. Liu , H. Wang , C. Lei , Y. Mao , H. Wang , X. He , X. Liang , Energy Storage Mater. 2022, 53, 544.

[43] J. Yang , D. Cai , Q. Lin , X. Wang , Z. Fang , L. Huang , Z. Wang , X. Hao , S. Zhao , J. Li , Nano Energy 2022, 91, 106669.

[44] D. Lin , Y. Li , Adv. Mater. 2022, 34, 2108856. 10.1002/adma.202108856 35119150

[45] Q. Guo , H. Wang , X. Sun , Y. N. Yang , N. Chen , L. Qu , ACS Mater. Lett. 2022, 4, 1872.

[46] J. Wang , J. Polleux , J. Lim , B. Dunn , J. Phys. Chem. C 2007, 111, 14925.

[47] H. Kim , J. Cook , H. Lin , J. S. Ko , S. H. Tolbert , V. Ozolins , B. Dunn , Nat. Mater. 2017, 16, 454. 27918566 10.1038/nmat4810

[48] Z. Su , Z. Wei , C. Lai , H. Deng , Z. Liu , J. Ma , Energy Storage Mater. 2018, 14, 129.

