# Supplementary material for: Polyoxometalate‐Bridged Synthesis of Superstructured Mesoporous Polymers and Their Derivatives for Sodium–Iodine Batteries
Source: Adv Sci (Weinh). 2023 Apr 25;10(19):2301918. doi: 10.1002/advs.202301918 (PMC10323648; doi:10.1002/advs.202301918)
Supplement: Supplementary file 1 — Supporting Information [file ADVS-10-2301918-s001.pdf]

ADVANCED SCIENCE
Open Access

Supporting Information

for *Adv. Sci.*, DOI 10.1002/advs.202301918

Polyoxometalate-Bridged Synthesis of Superstructured Mesoporous Polymers and Their Derivatives for Sodium–Iodine Batteries

*Tingting Zhang, Facai Wei, Yong Wu, Wenda Li, Lingyan Huang, Jianwei Fu, Chengbin Jing, Jiangong Cheng and Shaohua Liu**

Supporting Information

Polyoxometalate-Bridged Synthesis of Superstructured Mesoporous Polymers and Their Derivatives for Sodium-Iodine Batteries

*Tingting Zhang[a], Facai Wei[a], Wenda Li[a], Yong Wu[a], Lingyan Huang[a], Chengbin Jing[a], Jianwei Fu[b], Jiangong Cheng[c], Shaohua Liu[a]**

Experimental Procedures

Chemical Reagents and Materials

p-Phenylenediamine (PD, 99%, RG), $(NH_4)_6Mo_7O_{24}$ • $4H_2O$ (99%, RG)， $H_4[Si(W_3O_{10})_4]$ • xH_2O (AR), $Na_3O_{40}PW_{12}$ • xH_2O (99%, RG), and H_3PO_4 • $12MoO_3$ (98%, RG), Toluene, and Iodine (I_2) were purchased from Adamas Reagent (Shanghai). Triblock copolymer Pluronic F127 ($PEO_{106}PPO_{70}PEO_{106}$, Mw = 12600 g mol^{-1}), polyvinylidene fluoride (PVDF), and N-methyl-2-pyrrolidone (NMP) were obtained from Sigma-Aldrich Co., Ammonium persulfate (APS) was purchased from MACKLIN reagent. Ethanol (99.7%, AR) was purchased from General-reagent®. Deionized water was obtained from Millipore Autopure system and used in all experiments. All the reagents were used directly without any purification.

Characterizations and Methods

Scanning electron microscopy (SEM) images were obtained by Zeiss Gemini 450. Transmission electron microscopy (TEM) images were obtained by a JEM-2100F microscope with a field emission gun operated at 200 kV. The TEM samples were prepared by dropping sample dispersion on a carbon-supported copper grid and dried for 1 hour at room temperature. X-ray diffraction:(XRD) data of powder was collected on an Empyrean X-ray diffractometer with Cu Kα radiation (λ = 1.54056 Å, 40 kV, 40 mA). The scanning ranges from 5° to 80° with scanning rate of 10° min^{-1}. Fourier transform infrared (FTIR) spectra were performed on Nicolet iS50 spectrometer (Thermo Fisher) ranging from 4000 cm^{-1} to 500 cm^{-1}. Nitrogen absorption-desorption measurements were conducted on a Quantachrome Quadrasorb SI instrument after the sample was degassed at 120 °C overnight before actual measurement. The specific surface area was calculated by the Brunauer-Emmett-Teller (BET) method and the pore size distribution was derived from Barrett-Joyner-Halenda (BJH) method. X-ray photoelectron spectroscopy (XPS) measurements were performed under the pressure of $5 * 10^{-7}$ mbar by using a Thermo Fisher X-ray photoelectron spectrometer system equipped with an Al radiation probe (Thermo Scientific K-Alpha ESCALAB 250 Xi USA). Raman spectra were recorded on confocal micro-Raman spectrometer (Jobin-Yvon LabRAM HR Evolution, Horiba) with the excitation laser of 532 nm. UV-visible spectra were recorded by a Shimadzu UV- 2600 spectrophotometer. Zeta potential values were conducted on Zetasizer Nano ZS analyzer.

Experimental details

Synthesis of Mo-mPPD sample.

In a typical synthesis, 100 mg F127 was firstly dissolved in 10 mL of mixture solution (5 mL of water and 5 mL of ethanol) in a glass vail. The solution was stirred vigorously for 1 h to generate the micellar aggregates.

Then 200 μL of toluene was added to forming an emulsion solution. Afterwards, 1 mL of PD solution (0.1M in aqueous solution) and 500 μL of AMT solution (0.08M in aqueous solution) were sequentially added to the mixed solution and kept stirring for 2 h. Then 500 μL of APS (1M in aqueous solution) was added to initiate PD monomer polymerization. After reacting for 8 hours, MoO_x^{2-}/mesoPPD particles were obtained through centrifugation and washed with DI water and ethanol for several times. The final products were dried under vacuum at 60 °C for 12 h. Using the same procedure, $H_4[Si(W_3O_{10})_4] \cdot xH_2O$, $Na_3O_{40}PW_{12} \cdot xH_2O$, and $H_3PO_4 \cdot 12MoO_3$ were selected to replace AMT at the same concentration to obtain the corresponding products. The nonporous Mo-PPD samples were synthesized under the same procedure without adding toluene.

Synthesis of γ-Mo_2N/ mNC and γ-Mo_2N/NC samples.

The obtained Mo-mPPD and nonporous Mo-PPD samples were heated under nirogen atmosphere at 350 ° for 2 h and 900 °C for 2 h with a temperature-rising rate of 5 °C min^{-1}, γ -Mo_2N/ mNC and γ -Mo_2N/NC were obtained respectively.

Synthesis of NC sample.

100 mg PD monomer was dissolved into 5 mL DI water, and 500 mg APS was added subsequently to induce the polymerization of PD. The obtained product of poly(p-phenyldiamine) was washed by water and ethanol for at least 3 times. Then corresponding NC was obtained under the same heat treatment procedure.

Synthesis of I_2@γ-Mo_2N/mNC and I_2@γ-Mo_2N/NC samples.

The preparation of I_2@γ-Mo_2N/ mNC and I_2@γ-Mo_2N/NC through a same sublimation diffusion method. I_2 (400 mg) was spread on the bottom of a sealed reactor, and then 50 mg powders in a 5 mL glass vial were placed in the sealed reactor. The sealed reactor was heated to 180 °C for 12 h. The specific I_2 content of the composite was calculated based on the mass difference of samples before and after I_2 sublimation diffusion.

Electrochemical Measurements

The working electrodes were prepared by mixing the obtained active material samples, PVDF binder and Super P carbon black (Nanjing XFNANO Materials Tech Co., Ltd) with a mass ratio of 7:2:1 using NMP solvent. The above slurry was evenly casted on carbon cloth with a diameter of 12 mm and then dried at 60 °C for 12 h. The mass loading of active materials on the electrodes is in the range of 0.7-1.5 mg cm^{-2}. The 2032-type coin cells were assembled in an Ar-filled glove box (O_2 < 0.1 ppm and H_2O < 0.1 ppm). 1M $NaClO_4$ in ethylene carbonate and diethyl carbonate (EC/DEC) (1:1 by volume) with 5% 4-Fluoroethylene Carbonate (FEC) was used as electrolyte (DoDoChem, Suzhou, China). Na foil was utilized as reference/counter

electrode and glass fiber membranes (Whatman® GF/C) was used as the separator. Cyclic voltammetry (CV) tests were performed on an electrochemical workstation (CHI760E, Chenhua, Shanghai). Galvanostatic charge/discharge measurements were carried out at various current densities using a battery testing system (LAND CT2001A) at a voltage range of 1.5-3.3 V (vs Na^+/Na). All specific capacities and current densities are normalized to the mass of iodine.

Density Functional Theory (DFT) Calculations

The crystals were performed using the Cambridge Sequential Total Energy Package (CASTEP)1 based on the pseudopotential plane wave (PPW) method. [1] Electron-ion interactions were described using the ultrasoft (USP) potentials. A plane-wave basis set was employed to expand the wave functions with a cutoff kinetic energy of 400 eV. For the electron-electron exchange and correlation interactions, the functional parametrized by Perdew-Burke-Ernzerhof (PBE), a form of the general gradient approximation (GGA), was used throughout. The convergence criterion for the electronic self-consistent field (SCF) loop was set to 2×10-6 eV/atom. The atomic structures were optimized until the residual forces were below 0.05 eVÅ-1. The adsorption energy of AB is calculated as follows:

$$E_{ads}(A) = E(*A) - E(*) - E(A).$$

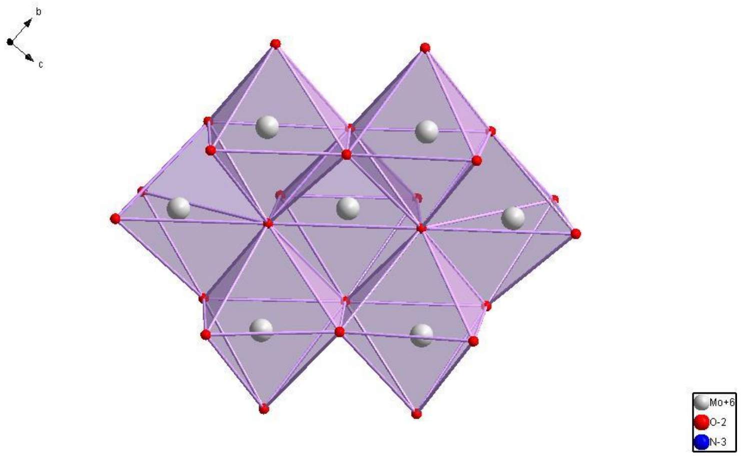

Figure S1. Schematic diagram of the crystal structure for homo-polyacids $Mo_7O_{24}^{6-}$.

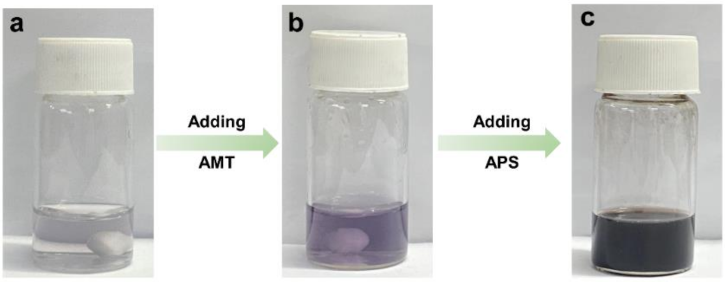

Figure S2. Photographs of mixed solutions under continuous stirring for (a) PD/micelle, (b) PD/micelle/AMT, (c) PD/micelle/AMT/ APS.

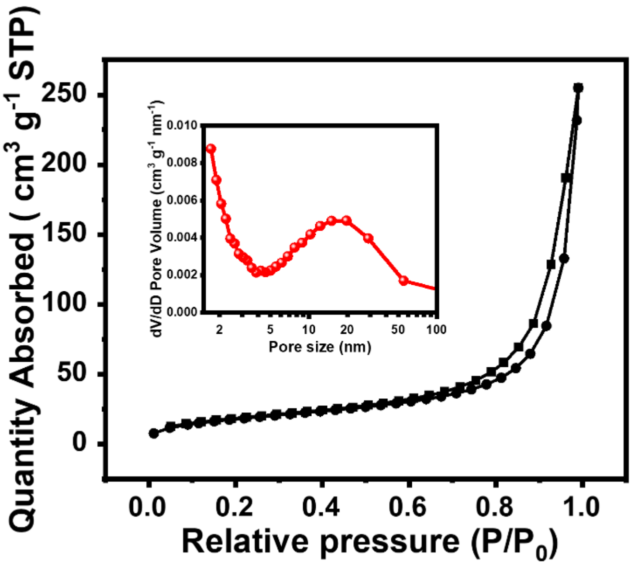

Figure S3. Nitrogen isotherm absorption curve and pore size distribution (inset) of Mo-mPPD sample calculated by BJH method.

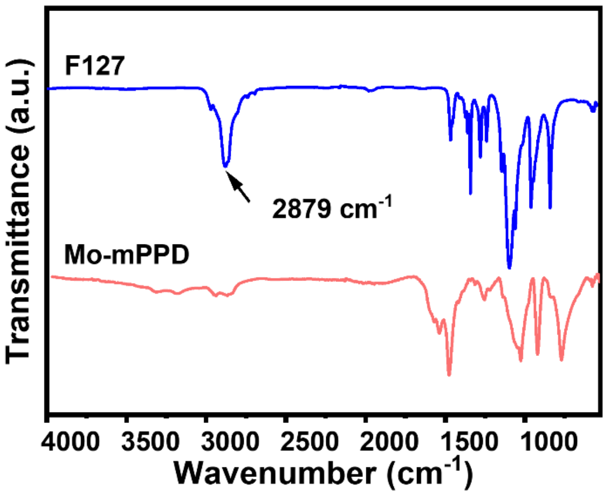

Figure S4. FT-IR spectra of F127 and Mo-mPPD samples.

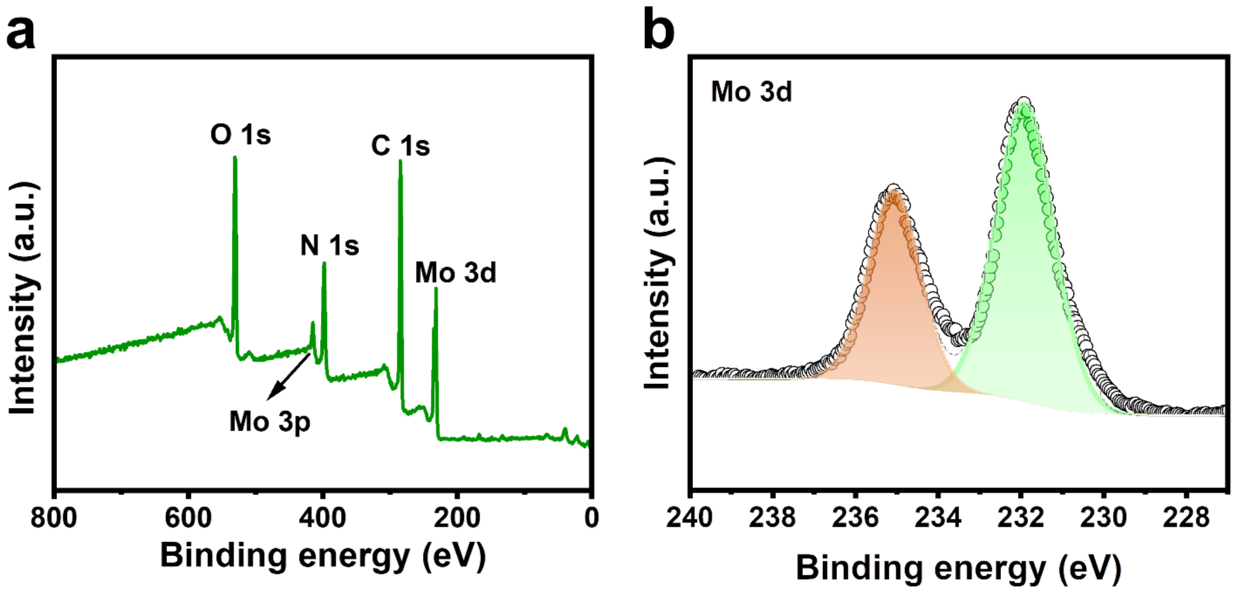

Figure S5. (a) XPS full spectrum, and high resolution Mo 3d XPS spectra (b) of Mo-mPPD sample.

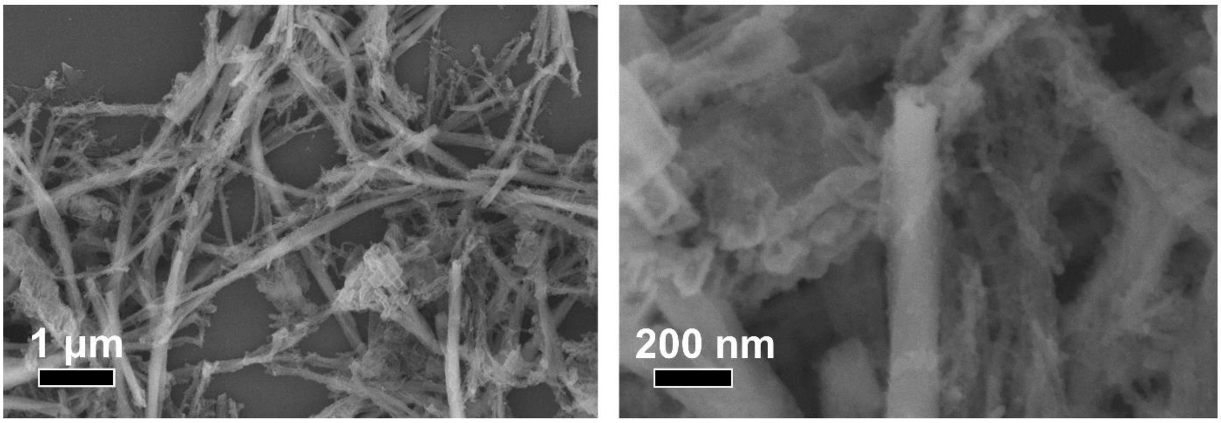

Figure S6：SEM images of PPD sample prepared without adding F127.

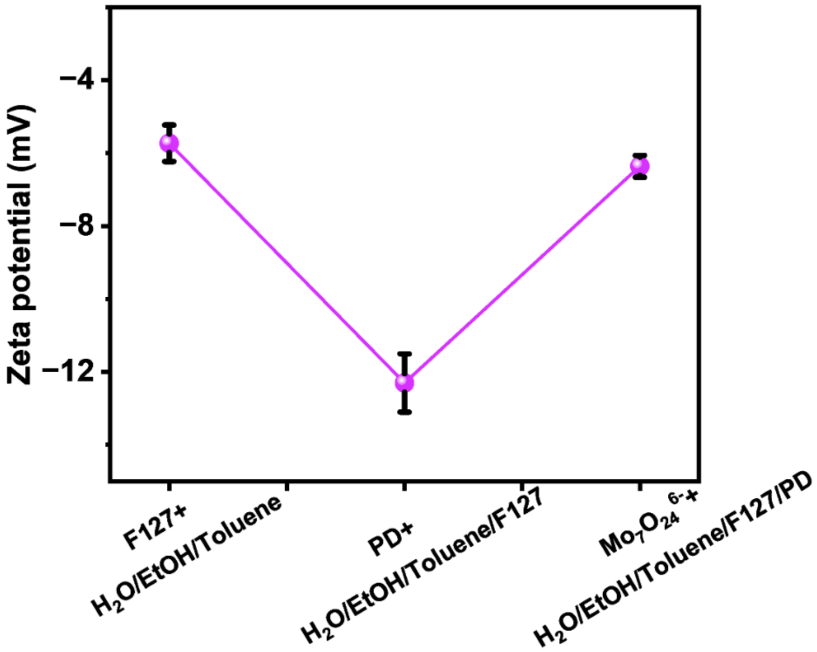

Figure S7. Zeta potential values of different mixed solution.

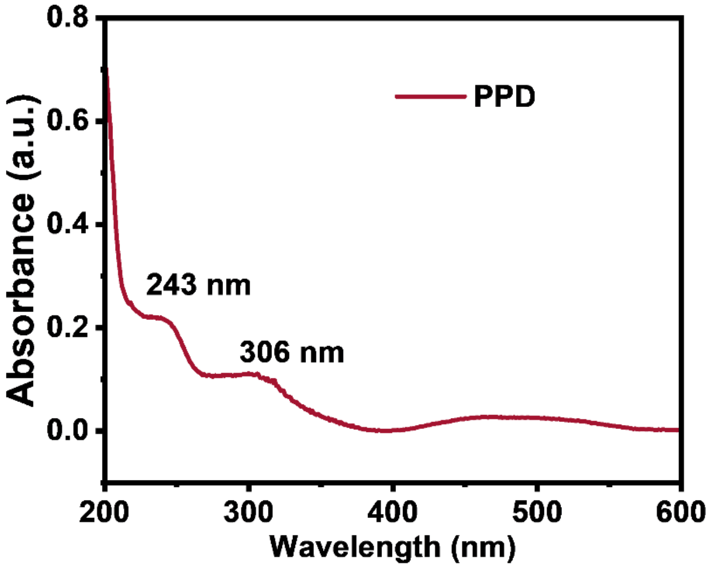

Figure S8. UV-Vis absorption spectrum of PPD.

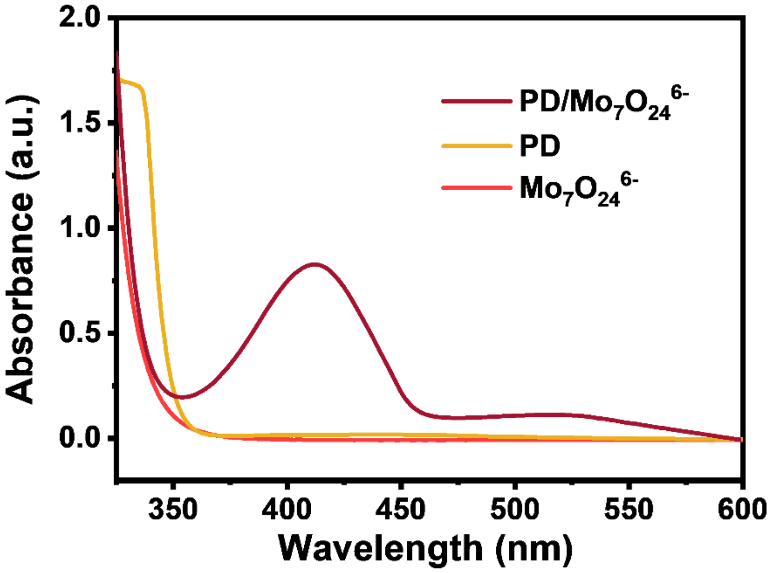

Figure S9. UV-Vis absorption spectra of PD, $(NH_4)_6Mo_7O_{24}\cdot 4H_2O$, and PD/$Mo_7O_{24}^{6-}$ mixed solution.

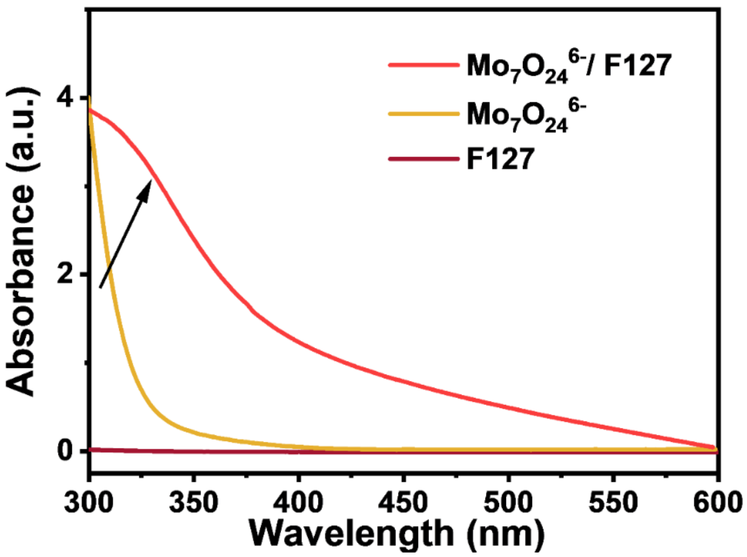

Figure S10. UV-Vis absorption spectra of F127 micelle solution, $(NH_4)_6Mo_7O_{24}\cdot4H_2O$, and $Mo_7O_{24}^{6-}$/F127 mixed solution.

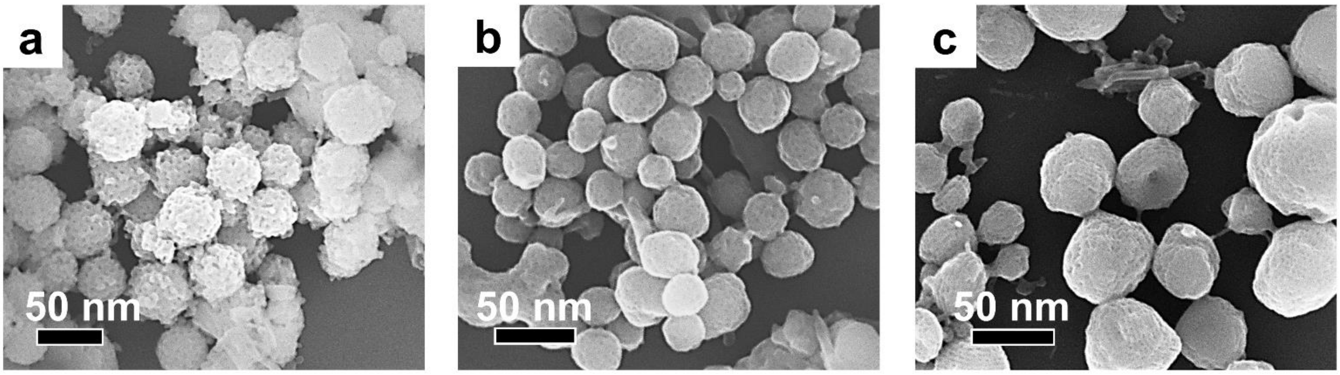

Figure S11. SEM images of hybrid samples synthesized by adding various POMs, (a) $Na_3O_{40}PW_{12}\cdot xH_2O$, (b) $H_3PO_4\cdot 12MoO_3$, and (c) $H_4[Si(W_3O_{10})_4]\cdot xH_2O$.

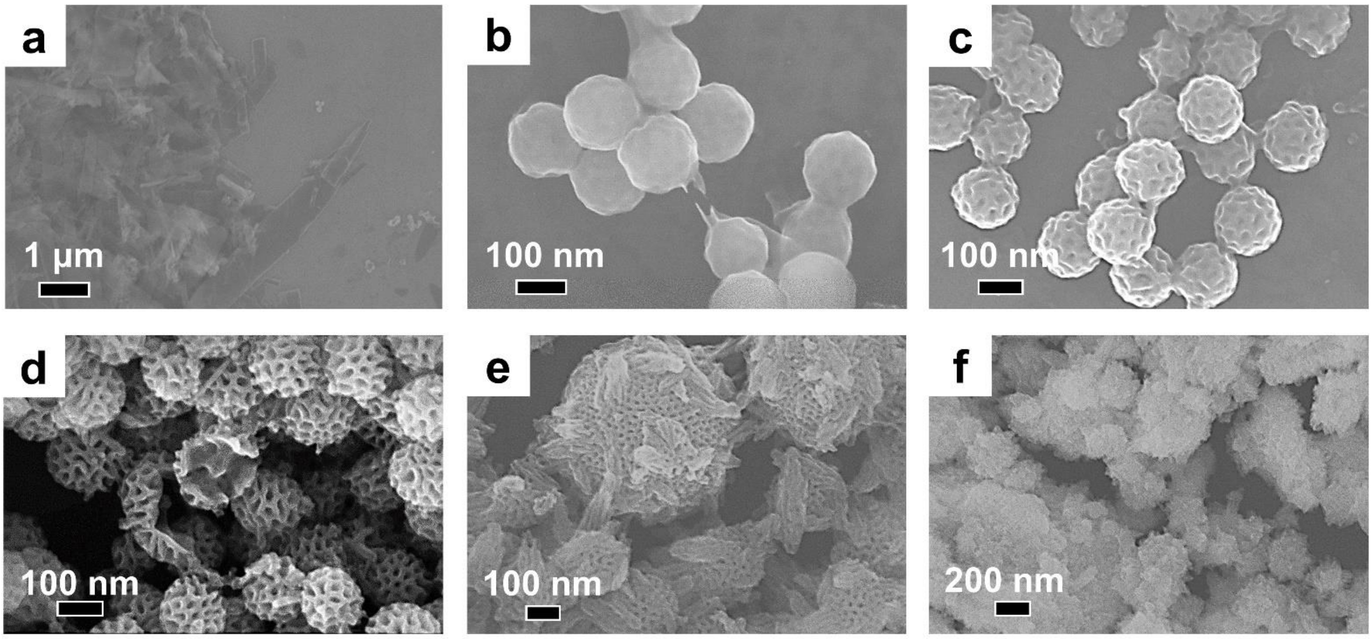

Figure S12. SEM images of PPD particles prepared by different PD / AMT molar ratios in the same F127 micelle solution: (a) 22:1, (b)12:1, (c) 11:1, (d) 5.5:1, (e) 3:1, and (f)1:1. With the molar ratio of PD/AMT decreases to 12:1, the morphology of mesoporous nanospheres appeared. While the molar ratio comes to 3:1, the nanosphere transformed into irregular particles, which can be ascribed to strong coordination between PD and AMT, destroying nano-emulsion assembly.

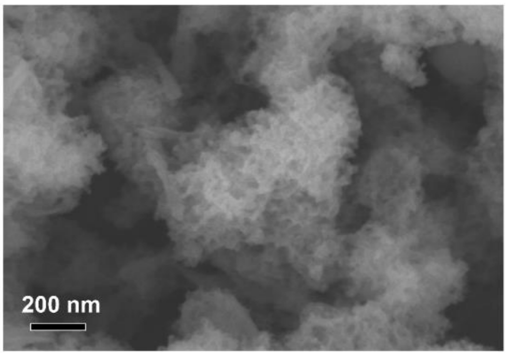

Figure S13. SEM images of PPD particles prepared without F127 in the same mixed solution.

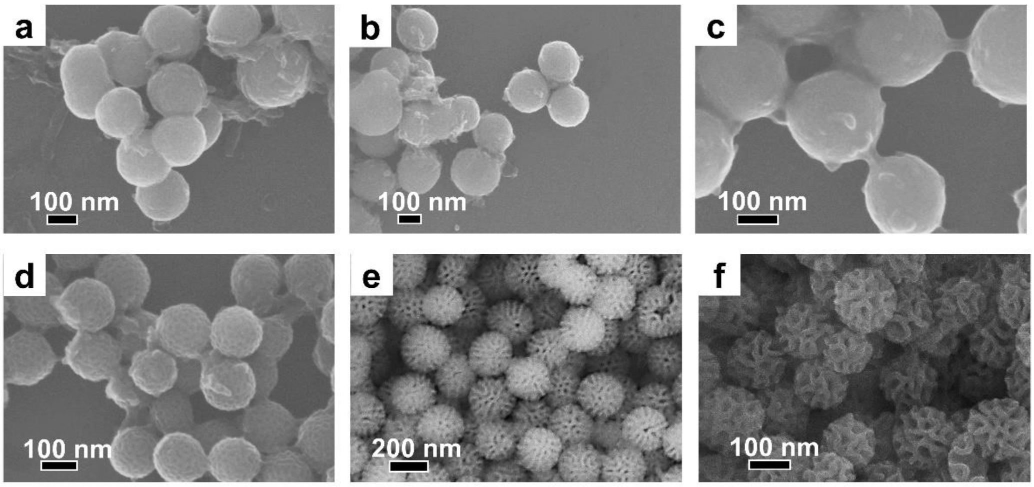

Figure S14. SEM images of Mo-mPPD samples prepared using a similar procedure by varying toluene/F127 mass ratios: (a) 0, (b) 0.5, (c) 1, (d)1.5, (e) 2, and (f) 2.5.

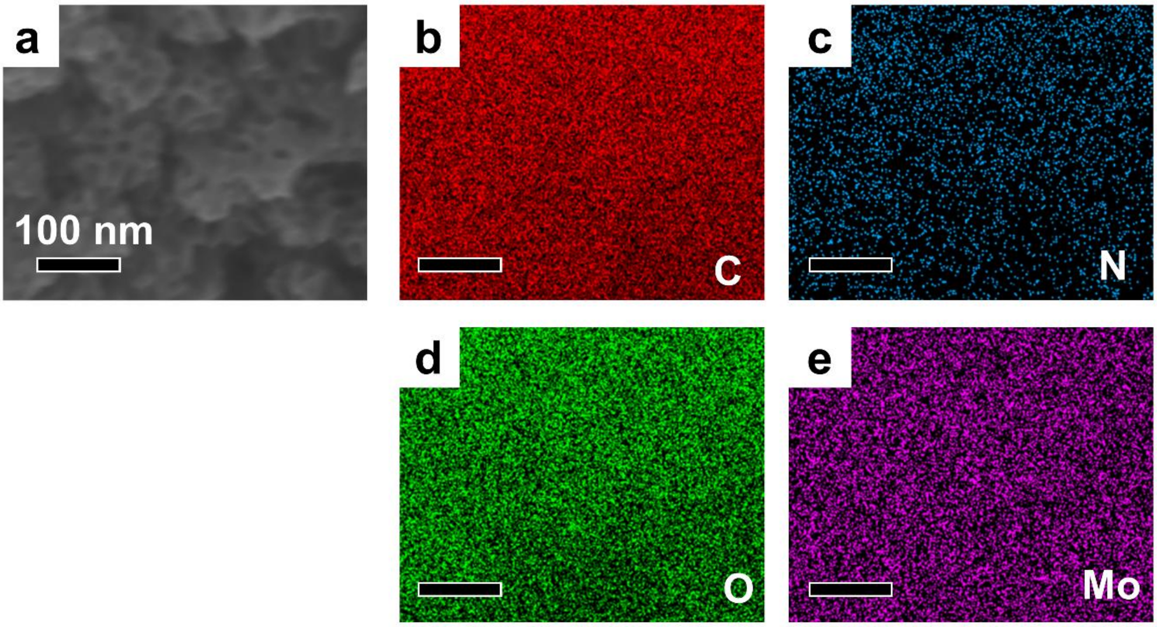

Figure S15. (a) SEM image and (b-e) corresponding elemental mapping images of γ-Mo_2N/mNC samples.

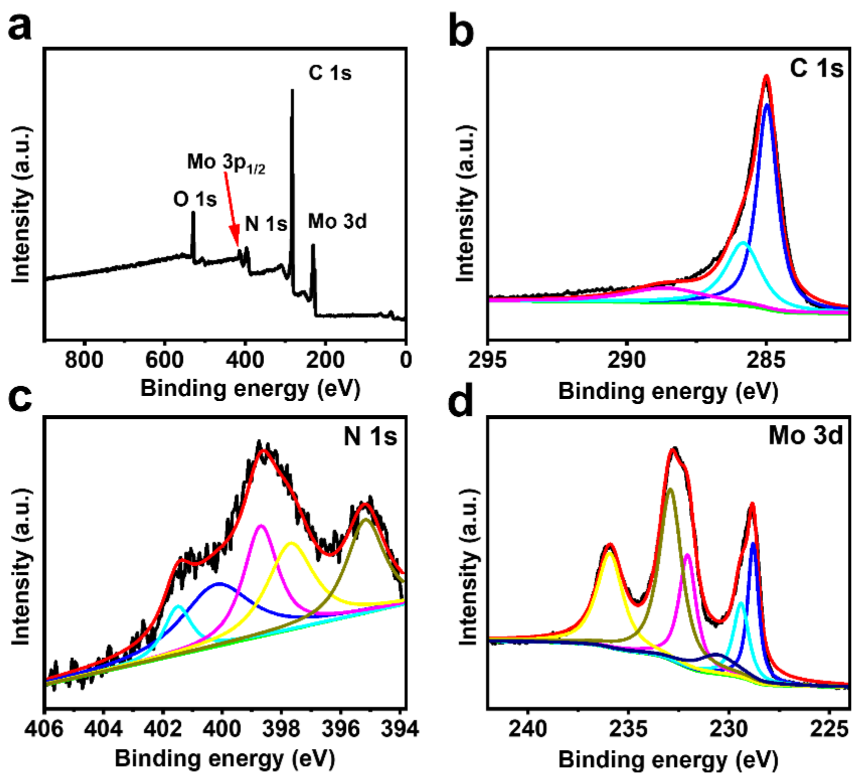

Figure S16. (a) XPS full spectrum and the high resolution XPS peaks of (b) C 1s, (c) N 1s, and (d) Mo 3d of γ-Mo_2N/mNC sample.

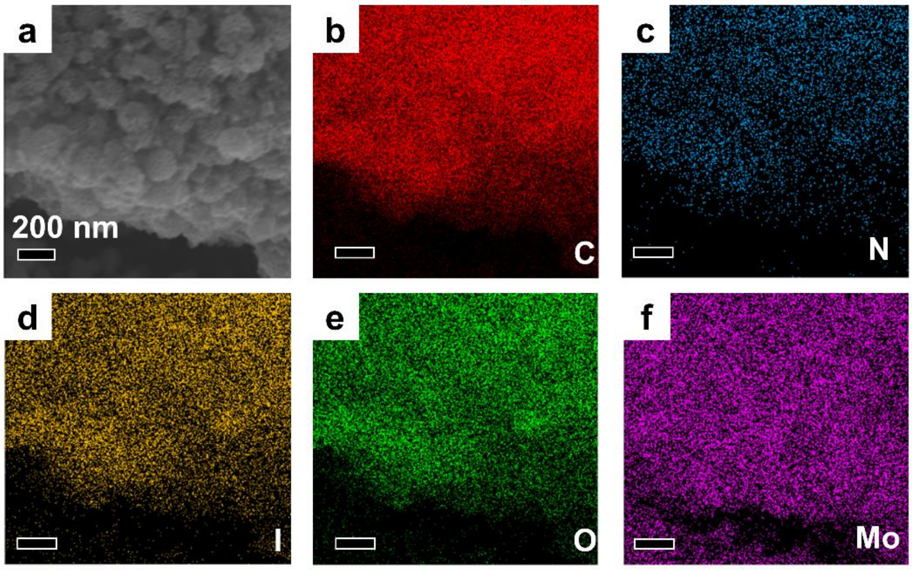

Figure S17. (a) SEM image and (b-e) corresponding elemental mapping of I_2@γ-Mo_2N/mNC sample.

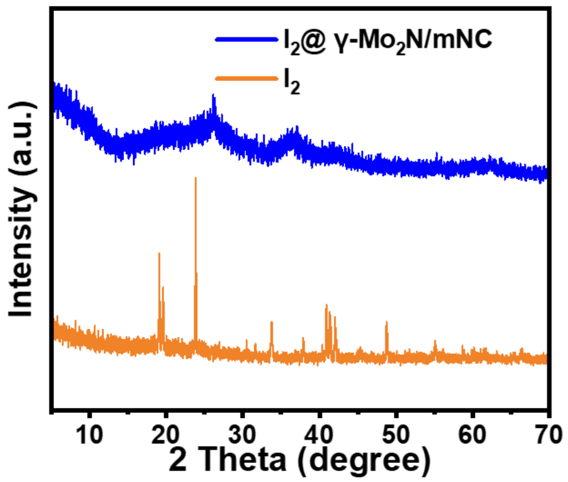

Figure S18. XRD patterns of I_2 and I_2@γ-Mo_2N/mNC samples.

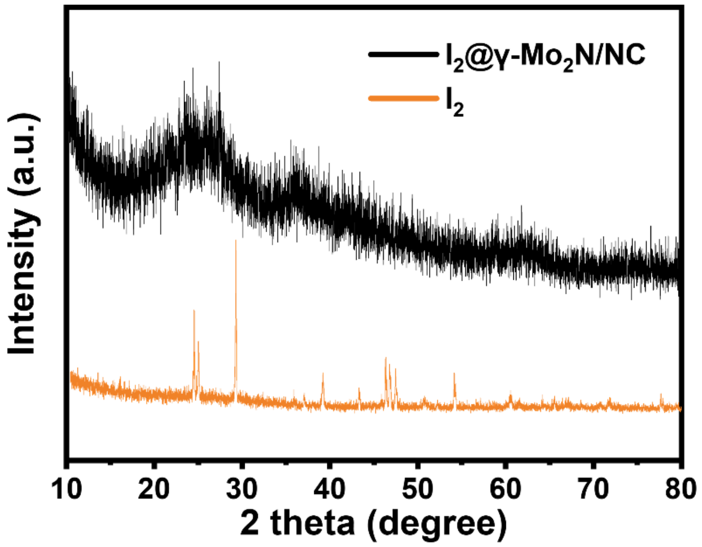

77

Figure S19. XRD patterns of I_2 and of I_2@γ-Mo_2N/NC samples.

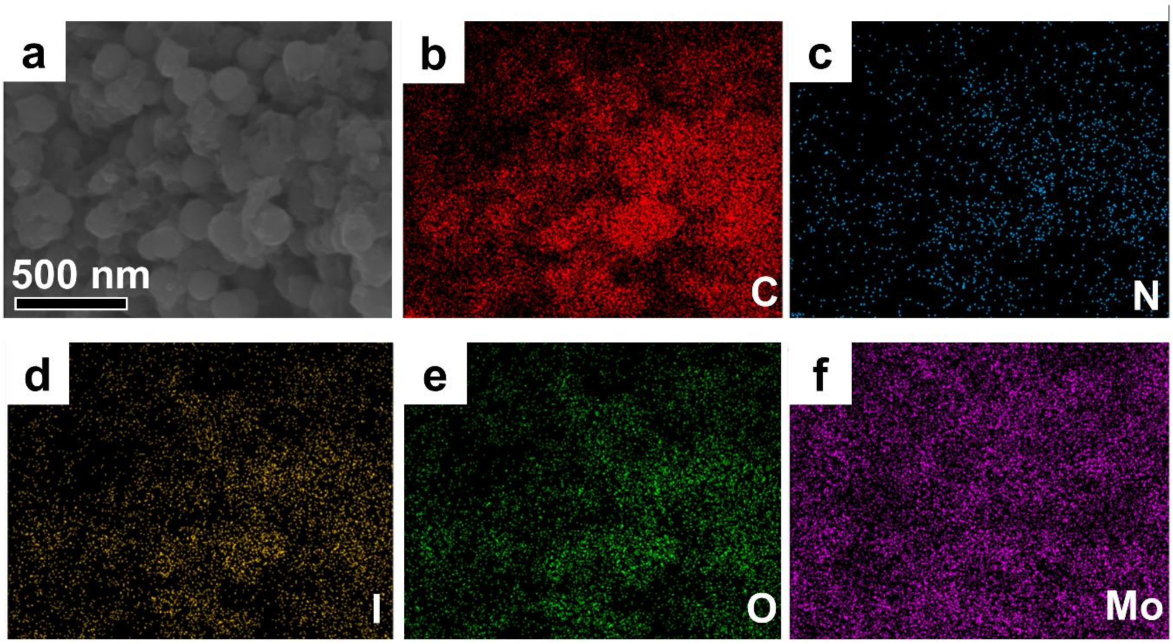

Figure S20. (a) SEM image and (b-e) corresponding elemental mapping of I_2@γ-Mo_2N/NC sample.

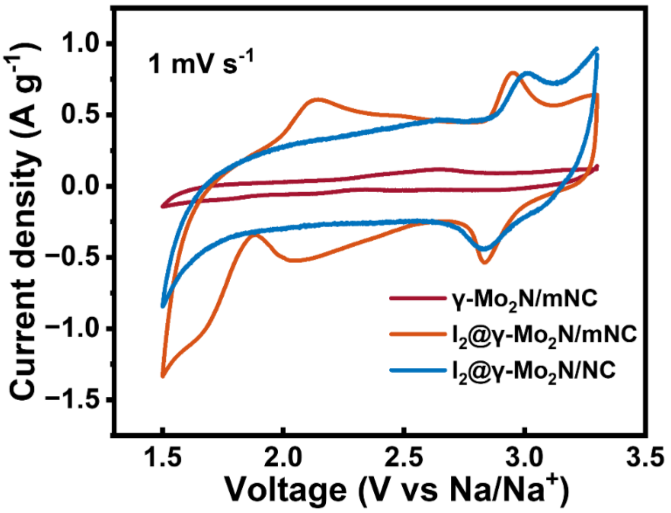

Figure S21. CV curves of I_2@γ-Mo_2N/mNC, I_2@γ-Mo_2N/NC, and γ-Mo_2N/mNC electrodes at 1 mV s^{-1}.

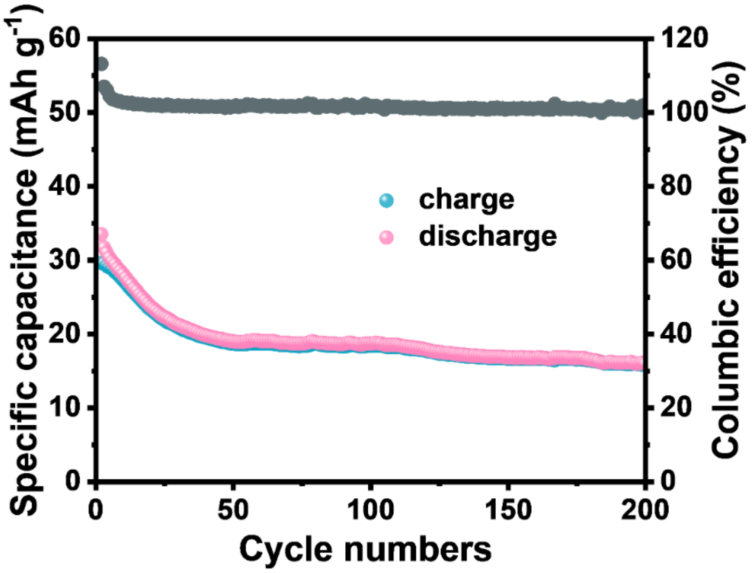

Figure S22. Cycling performance of γ-Mo_2N/mNC electrode at 0.75 A g^{-1}.

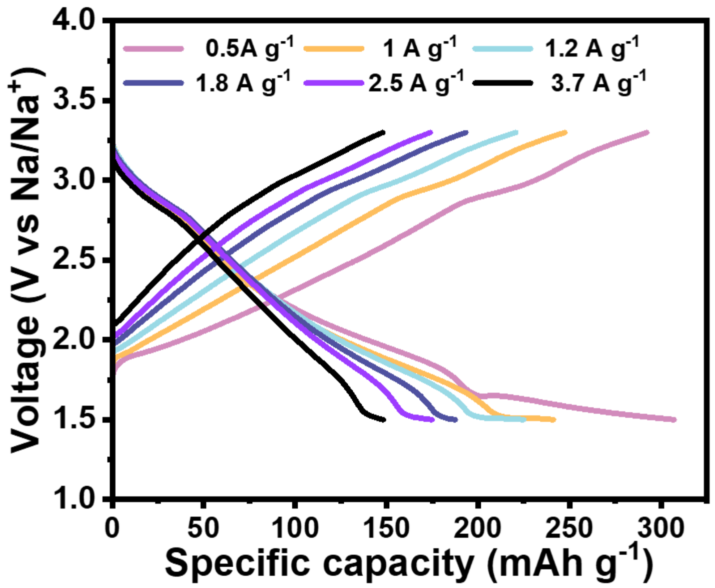

Figure S23. Discharge and charge curves of I_2@γ-Mo_2N/mNC electrode at 0.5 A g^{-1}, 1 A g^{-1}, 1.2 A g^{-1}, 1.8 A g^{-1}, 2.5A g^{-1}, and 3.7 A g^{-1}.

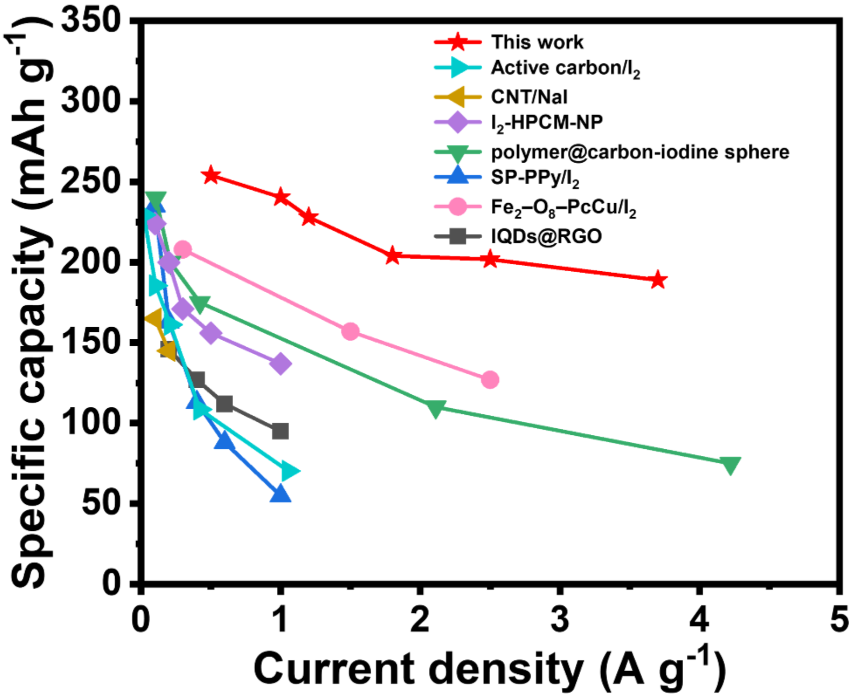

88

Figure S24. Comparison of Na-I_2 battery with other published works (SP-PPy/I_2,[2] Fe_2-O_8-PcCU/I_2,[3] IQDs@RGO,[4] Polymer@C-I_2 sphere,[5] I_2-HPCM-NP[6], CNT/NaI,[7] and Active carbon cloth/I_2[8]).

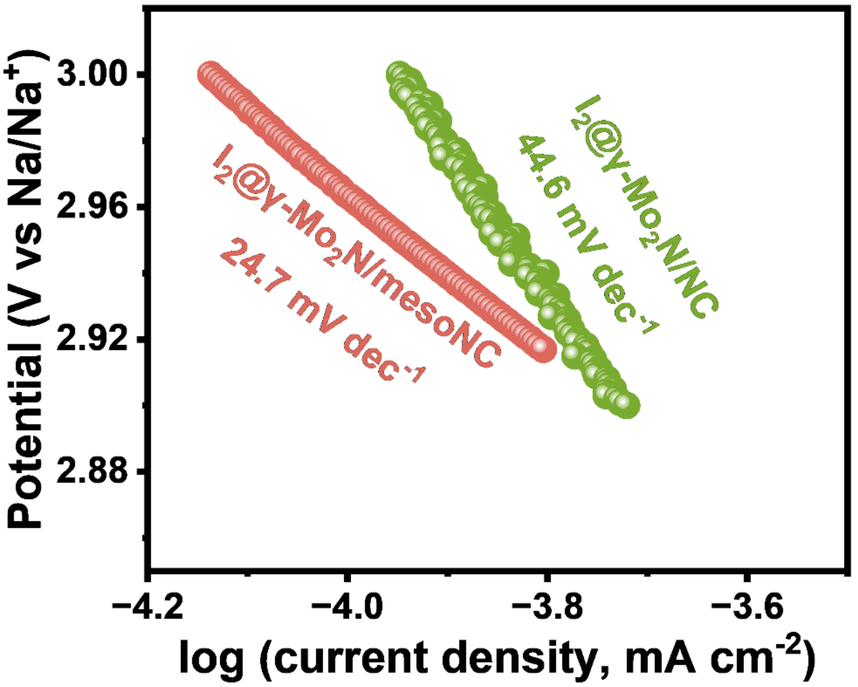

Figure S25. Tafel plots of I_2@γ-Mo_2N/mNC and I_2@γ-Mo_2N/NC electrodes. The derivation of the Tafel slope (η) from the CV curve provides a key parameter for characterizing the kinetics of a specific reaction and dominating the effect of electrocatalysis on the charge transfer kinetics during the Na-I_2 redox conversion. A smaller η means a faster reaction kinetics. It shows that the I_2@γ-Mo_2N/mNC electrode exhibits the smallest Tafel slope during the anodic scan (discharge 24 mV dec^{-1}), which is lower than that of the I_2@γ-Mo_2N/NC electrode (discharge 56 mV dec^{-1}). This further indicates that the I_2@γ-Mo_2N/mNC electrode reaction is greatly accelerated and exhibits better catalytic activity.

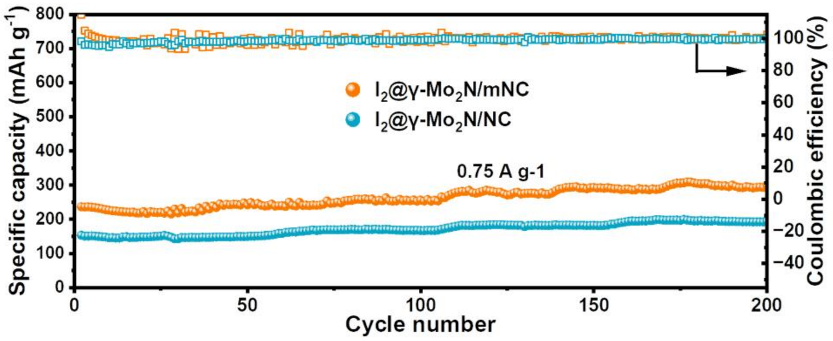

Figure S26.The cycle performance of I_2@γ-Mo_2N/mNC and I_2@γ-Mo_2N/NC electrodes.

Table S1. The element percentages of the Mo-mPPD and γ-Mo_2N/mNC samples obtained from XPS results.

	C (At%)	N (At%)	O (At%)	Mo (At%)
Mo-mPPD	56.23	19.53	21.18	3.06
γ -Mo_2N/mNC	79.27	6.75	9.74	4.23

Table S2. Comparison of Na-I_2 battery with other published works.

**Cathode materials**	**Anode materials**	**I_2 weight content (%)**	**Rate (mA g^1)**	**Specific Capacity (mAh g^{-1})**	**Ref.**
**γ-Mo_2N/mNC**	**Na**	**25~30**	**500**	**254**	**This work**
SP-PPy/I_2	Na	46	105.5	235	[2]
Fe_2-O_8-PcCu/I_2	Na	40	300	208	[3]
IQDs/RGO	Na	26	100	170	[4]
Polymer@C-I_2 sphere	Na	36	105.5	190	[5]
I_2-HPCM-NP	Na	40	100	224	[6]
I_2-HPCM-NP	Na	NaI (25-30%)	100	165	[7]
CNT/NaI	Na-NaI	/	105.5	185.5	[8]

References

[1] Accelerys Materials Studio Release Notes, Release 5.5.1; Accelrys Software, Inc.: San Diego, 2010.

[2] L. Xiang, S. Yuan, F. Wang, Z. Xu, X. Li, F. Tian, L. Wu, W. Yu, Y. Mai, *J. Am. Chem. Soc.* **2022**, 144, 15497.

[3] F. Wang, Z. Liu, C. Yang, H. Zhong, G. Nam, P. Zhang, R. Dong, Y. Wu, J. Cho, J. Zhang, *Adv. Mater* .**2020**, 32, 1905361.

[4] D. Gong, B. Wang, J. Zhu, R. Podila, A. M. Rao, X. Yu, Z. Xu, B. Lu, *Adv. Energy Mater.* **2017**, 7, 1601885.

[5] G. Zhang, H. Wang, S. Zhang, C. Deng, *J Mater. Chem.A* **2018**, 6, 9019.

[6] K. Lu, Z. Hu, J. Ma, H. Ma, L. Dai, J. Zhang, *Nat. Commun.* **2017**, 8, 527.

[7] S. Kim, X. Li, L. Sang, Y. S. Yun, R. G. Nuzzo, A. A. Gewirth, P. V. Braun, *Adv. Mater. Interfaces* **2018**, 5, 1801342.

[8] H. Tian, H. Shao, Y. Chen, X. Fang, P. Xiong, B. Sun, P. H. L. Notten, G. Wang, *Nano Energy* **2019**, 57, 692.
